# Proteomic Analysis Reveals the Leaf Color Regulation Mechanism in Chimera *Hosta* “Gold Standard” Leaves

**DOI:** 10.3390/ijms17030346

**Published:** 2016-03-08

**Authors:** Juanjuan Yu, Jinzheng Zhang, Qi Zhao, Yuelu Liu, Sixue Chen, Hongliang Guo, Lei Shi, Shaojun Dai

**Affiliations:** 1 Development Center of Plant Germplasm Resources, College of Life and Environmental Sciences, Shanghai Normal University, Shanghai 200234, China; yujuan8186@163.com (J.Y.); zhaoqizq@yeah.net (Q.Z.); 2 Alkali Soil Natural Environmental Science Center, Northeast Forestry University, Key Laboratory of Saline-alkali Vegetation Ecology Restoration in Oil Field, Ministry of Education, Harbin 150040, China; 3 Institute of Botany, Chinese Academy of Sciences, Beijing 100093, China; caohua@ibcas.ac.cn (J.Z.); liuyuelu09@sina.com (Y.L.); shilei@ibcas.ac.cn (L.S.); 4 Department of Biology, Genetics Institute, Plant Molecular and Cellular Biology Program, Interdisciplinary Center for Biotechnology Research, University of Florida, Gainesville, FL 32610, USA; schen@ufl.edu; 5 Food Engineering College, Harbin University of Commerce, Harbin 150028, China; guohongliang97@126.com

**Keywords:** *Hosta* “Gold Standard”, proteomics, variegated leaves, leaf color, excess nitrogen fertilization

## Abstract

Leaf color change of variegated leaves from chimera species is regulated by fine-tuned molecular mechanisms. *Hosta* “Gold Standard” is a typical chimera *Hosta* species with golden-green variegated leaves, which is an ideal material to investigate the molecular mechanisms of leaf variegation. In this study, the margin and center regions of young and mature leaves from *Hosta* “Gold Standard”, as well as the leaves from plants after excess nitrogen fertilization were studied using physiological and comparative proteomic approaches. We identified 31 differentially expressed proteins in various regions and development stages of variegated leaves. Some of them may be related to the leaf color regulation in *Hosta* “Gold Standard”. For example, cytosolic glutamine synthetase (GS1), heat shock protein 70 (Hsp70), and chloroplastic elongation factor G (cpEF-G) were involved in pigment-related nitrogen synthesis as well as protein synthesis and processing. By integrating the proteomics data with physiological results, we revealed the metabolic patterns of nitrogen metabolism, photosynthesis, energy supply, as well as chloroplast protein synthesis, import and processing in various leaf regions at different development stages. Additionally, chloroplast-localized proteoforms involved in nitrogen metabolism, photosynthesis and protein processing implied that post-translational modifications were crucial for leaf color regulation. These results provide new clues toward understanding the mechanisms of leaf color regulation in variegated leaves.

## 1. Introduction

Leaf color change during plant development and envrionment response is regulated by complicated and fine-tuned molecular mechanisms. Current understandings of leaf color regulatory mechanisms are mainly based on the investigation of leaf color mutants of model plants and crops, such as Arabidopsis [1,2,3], rice [4], and tomato [5], rather than natural variegated plants. Variegated plants are widely distributed, some of which are cultivated as ornamental plants. Variegated leaves usually consist of green and white/yellow sectors, which are useful resources for investigating chloroplast development and color changes [6]. The chloroplastic or genetic origin of leaf variegation [6], as well as albinism mechanism has been reported [7,8]. Most investigations were focused on the physiological analysis of the white region and green regions [3,9,10,11], development of *aurea* “golden” leaves, and characteristics of yellow-green variegated leaves [12,13,14,15,16,17,18].

Various colors of leaf variegation suggest that different regulatory pathways are responsible in the respective cases [10]. Nitrogen nutrients and light intensities are considered as the main factors for yellow-green variegated leaves and leaves of *aurea* “golden” varieties from some vascular plants. Excess nitrogen fertilization [19], deep shaded [20], and long-term low light illumination [21] lead to green coloration of yellow leaves/regions. The conditional yellow-green variegated leafed plants or *aurea* “golden” varieties have been investigated for chloroplast ultrastructure [19,21,22,23], construction and function of photosystem I (PSI) and photosystem II (PSII) [21,24,25], chlorophyll synthesis and degradation [21], and plastid regulated expression of nuclear *cab* genes [26,27,28].

The plant species in genus *Hosta*, belonging to the family Liliaceae, are exceedingly popular perennials in today’s gardens. In these species, chimera *Hosta* with variegated leaves has been widely cultivated [29]. *Hosta* “Gold Standard” is a typical chimera *Hosta* species. The margin of its ovate leaves is dark green, and the center of leaves appears light green in the spring and become progressively more golden towards early summer [30]. Excess nitrogen fertilization will turn the golden-green variegated leaves to whole green leaves of *Hosta* “Gold Standard” [19,30]. Therefore, *Hosta* “Gold Standard” is considered as an ideal material to investigate the molecular mechanisms of leaf variegation.

Proteomics approaches provide a high-throughput and systematic analysis of protein networks and molecular regulatory mechanisms of plant development and environmental response [31,32,33,34,35]. Proteomics has been applied to the investigation of leaf coloring mechanisms (albinism) [7,8]. Comparative proteomic studies based on two-dimensional gel electrophoresis (2-DE) have revealed 14 chloroplast proteins changed in the stage albinism line of winter wheat *FA85* as compared with the parent wheat *Aibian 1* [7], and 26 proteins in periodic albinism of White leaf No. 1 (a typical albino tea cultivar grown in China) [8]*.* These identified proteins are involved in metabolism of nitrogen metabolism, photosynthesis, carbon and energy metabolism, RNA processing, protein processing, signal transduction, and stress and defense response, indicating these physiological processes might play important roles in the albinism [7,8]. The proteomic profiles from various leaf color tissues provide new evidence for leaf color regulation in albinistic leaves, but the molecular mechanisms in golden-green variegated leaves are still to be investigated.

In this study, the margin and center regions of young and mature leaves from *Hosta* “Gold Standard”, as well as the leaves after excess nitrogen fertilization were analyzed using physiological and comparative proteomic approaches. Our results indicate that nitrogen metabolism regulation, photosynthesis and energy supply, chloroplast development, and chloroplast protein import/processing play crucial roles in leaf color changes in variegated leaves. The results provide novel insights into understanding the mechanisms of leaf color regulation in variegated leaves.

## 2. Results

### 2.1. Variegated Leaf Color Changes in Different Development Stages

**Figure 1:**
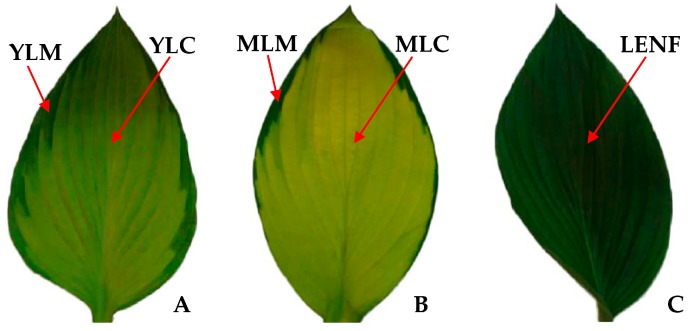
Representative leaves at different development stages of *Hosta* “Gold Standard”. (**A**) Young leaf; (**B**) mature leaf; (**C**) leaf under excess nitrogen fertilization. YLM: Young leaf margin; YLC: Young leaf center; MLM: Mature leaf margin; MLC: Mature leaf center; LENF: Leaf under excess nitrogen fertilization.

The variegated young leaves of *Hosta* “Gold Standard” seedlings were ovate with dark green margins and light green center (Figure 1A). During seedling development, the variegated mature leaves become progressively golden in leaf center, with the leaf margin kept dark green (Figure 1B). After 30 days of excess nitrogen fertilization, the whole leaves turned to green and the phenotype of the variegated leaf disappeared (Figure 1C).

**Figure 2:**
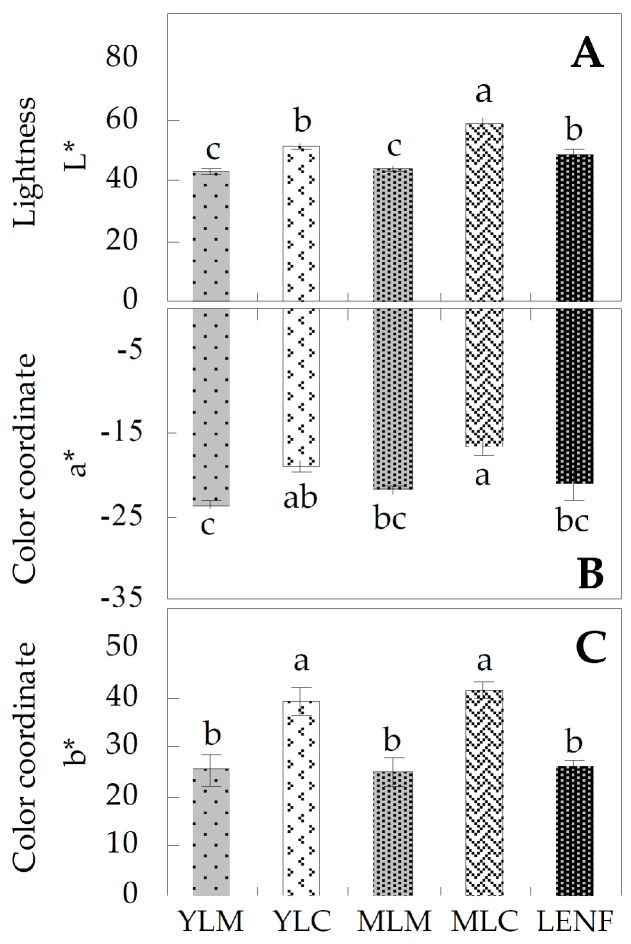
Color properties of various leaf regions at different development stages of *Hosta* “Gold Standard”. (**A**) Lightness L*, representing the perceived lightness; (**B**) color coordinate a*, indicating the change in hue from red to green; (**C**) color coordinate b*, indicating the change in hue from yellow to blue. The values were determined in young leaf margin (YLM), young leaf center (YLC), mature leaf margin (MLM), mature leaf center (MLC), and leaf under excess nitrogen fertilization (LENF), which were presented as means ± SE (*n* = 3). The different small letters on the columns indicate significant differences among various leaf regions at different development stages (*p* < 0.05).

The color properties of various leaf regions at different development stages of *Hosta* “Gold Standard” were determined. The value of lightness L* reflects the perceived lightness, and values of the color coordinate a* and b* represent the hue from red to green and from yellow to blue, respectively. There were significant differences in lightness L*, color coordinate a*, and color coordinate b* in the marginal regions and central regions either of both young leaves and mature leaves, indicating the variegated leaf phenotype (Figure 2). The lightness L*, color coordinate a*, and color coordinate b* did not have significant changes in mature leaf margin (MLM), when compared with the young leaf margin (YLM) (Figure 2). However, the color properties of the leaf center were changed during leaf development. Lightness L* increased obviously, although color coordinate a* and b* showed no obvious changes in mature leaf center (MLC) compared with young leaf center (YLC). After excess nitrogen fertilization for 30 days, lightness L*, color coordinate a*, and color coordinate b* were decreased significantly in leaves, when compared with MLC (Figure 2).

### 2.2. Chlorophyll Contents in Differrent Regions of Leaves

**Figure 3:**
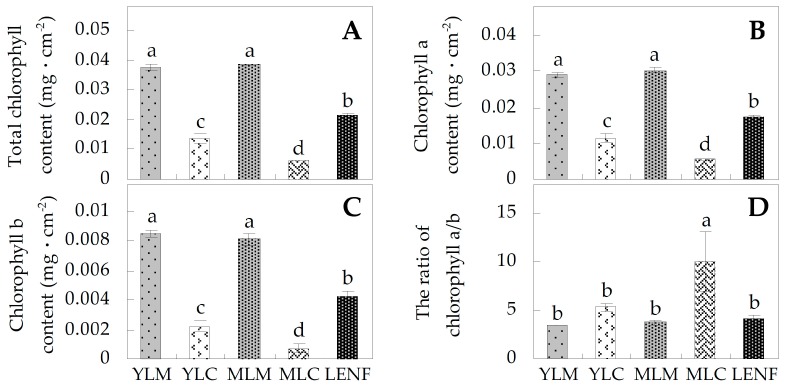
Chlorophyll contents of various leaf regions at different development stages of *Hosta* “Gold Standard”. (**A**) Total chlorophyll content; (**B**) chlorophyll *a* content; (**C**) chlorophyll *b* content; (**D**) the ratio of chlorophyll *a*/*b*. The values were determined in young leaf margin (YLM), young leaf center (YLC), mature leaf margin (MLM), mature leaf center (MLC), and leaf under excess nitrogen fertilization (LENF), which were presented as means ± SE (*n* = 3). The different small letters on the columns indicate significant differences among various leaf regions at different development stages (*p* < 0.05).

To evaluate the leaf color changes, the contents of chlorophylls in various regions of leaves were determined. The contents of total chlorophylls (Chls), chlorophyll *a* (Chl *a*), and chlorophyll *b* (Chl *b*) showed no significant changes in MLM compared with YLM (Figure 3A–C). But the contents of total Chls, Chl *a*, and Chl *b* were lower in the central regions than those in the marginal regions of young leaves and mature leaves, and the decrease of them was significant in mature leaves (Figure 3A–C). Besides, the ratio of Chl *a* to Chl *b* in MLC was the highest among various regions/stages (Figure 3D). After excess nitrogen fertilization for 30 days, the contents of total Chls, Chl *a* and Chl *b* in leaves under excess nitrogen fertilization (LENF) were all increased when compared with MLC, but they still were lower than MLM (Figure 3A–C).

### 2.3. Nitrogen Contents and Activities of Nitrogen Metabolism-Related Enzymes

**Figure 4:**
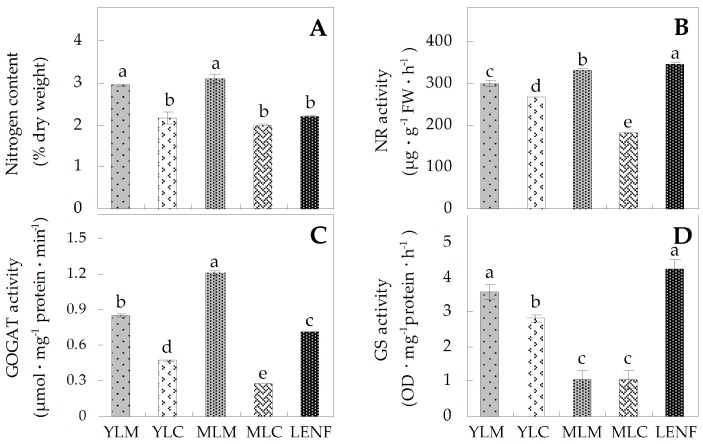
Nitrogen content and activities of nitrogen metabolism-related enzymes in various leaf regions at different development stages of *Hosta* “Gold Standard”. (**A**) Nitrogen content; (**B**) nitrate reductase (NR) activity; (**C**) glutamine α-oxoglutarate aminotransferase (GOGAT) activity; (**D**) glutamine synthetase (GS) activity. The values were determined in young leaf margin (YLM), young leaf center (YLC), mature leaf margin (MLM), mature leaf center (MLC), and leaf under excess nitrogen fertilization (LENF), which were presented as means ± SE (*n* = 3). The different small letters on the columns indicate significant differences among various leaf regions at different development stages (*p* < 0.05).

To monitor the nitrogen assimilation status in various leaf regions at different development stages from *Hosta* “Gold Standard” or under excess nitrogen fertilization, the nitrogen contents and the activities of nitrogen metabolism related enzymes were determined. During leaf development, the nitrogen contents in the centers of young leaves and mature leaves were all lower than in the margins of these leaves (Figure 4A). Accordingly, the activities of nitrate reductase (NR) and glutamine α-oxoglutarate aminotransferase (GOGAT) in YLC and MLC were reduced when compared with YLM and MLM (Figure 4B,C). The activities of glutamine synthetase (GS) in YLC were lower than YLM, but their activities in MLM and MLC were similar (Figure 4D). Interestingly, the nitrogen levels in LENF were similar with those in leaf centers and obviously lower than in the margins (Figure 4A). The activities of NR, GOGAT and GS in LENF were induced when compared with those in leaf centers, except the GOGAT activity was still lower than in leaf margins (Figure 4B–D).

### 2.4. Identification of Differentially Expressed Proteins (DEPs) in Various Leaf Regions

**Figure 5:**
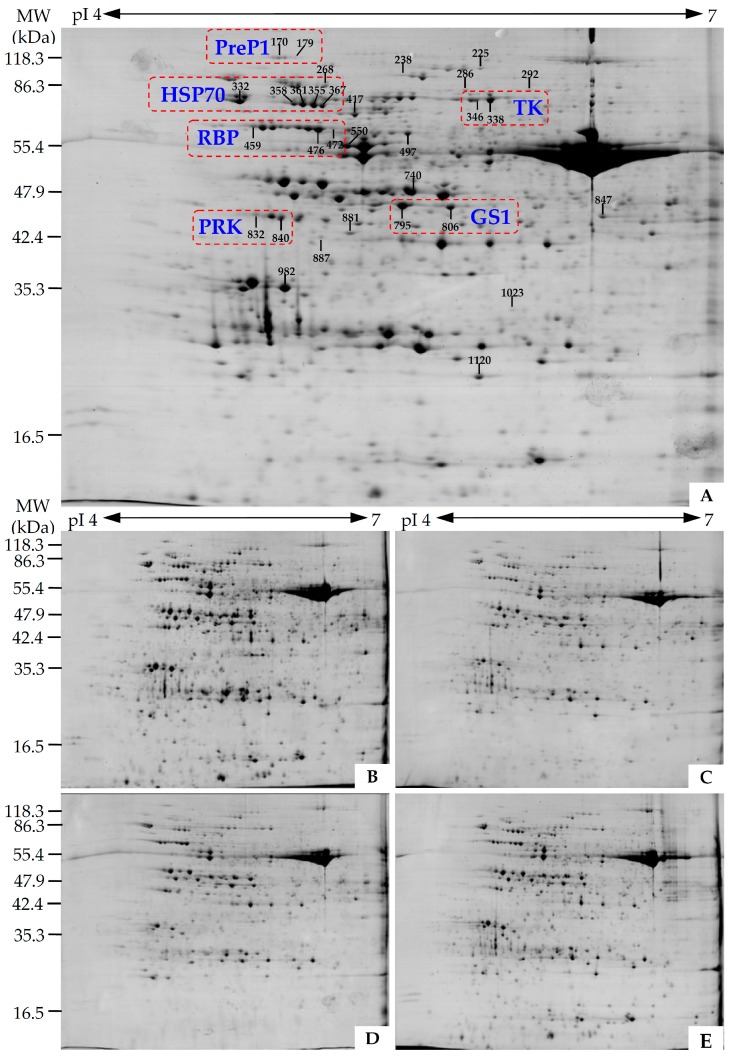
Representative 2-DE gel images of proteins extracted from various leaf regions at different development stages of *Hosta* “Gold Standard”. (**A**) Young leaf margin (YLM); (**B**) young leaf center (YLC); (**C**) mature leaf margin (MLM); (**D**) mature leaf center (MLC); (**E**) leaf under excess nitrogen fertilization (LENF). Proteins were separated on 24 cm immobilized pH gradient (IPG) strips (pH 4–7 linear gradient) using isoelectric focusing (IEF) in the first dimension, followed by 12.5% SDS-PAGE gels in the second dimension. The 2-DE gel was stained with Coomassie Brilliant Blue. Molecular weight (*M*w) in kilodaltons (kDa) and pI of proteins are indicated on the left and top of the gel, respectively. A total of 31 differentially expressed proteins identified by MALDI TOF-TOF MS were marked with numbers on the gel, and the detailed information can be found in Table 1, Supplemental Figure S1, and Supplemental Tables S1 and S2. The protein spots of proteoforms in each group were enclosed in a red rectangle. GS1: Glutamine synthetase isoform GS1c; Hsp70: Heat shock protein 70; PreP1: Presequence protease 1; PRK: Phosphoribulokinase; RBP: RuBisCO large subunit-binding protein subunit; TK, transketolase.

To investigate the DEPs in different leaf regions and nitrogen conditions, the protein profiles of five regions/stages of leaves (YLM, YLC, MLM, MLC, and LENF) were obtained using 2-DE analysis. On Coomassie Brilliant Blue-stained gels (24 cm immobilized pH gradient (IPG) strip, pH 4–7), 908 ± 15, 917 ± 39, 873 ± 30, 856 ± 53, and 839 ± 19 protein spots from YLM, YLC, MLM, MLC, and LENF were detected, respectively (Figure 5, Supplemental Figure S1). After gel image analysis on the basis of the calculated average vol % values of each matched protein spot, 42 reproducibly matched spots showed more than a 1.5-fold change in abundance (*p* < 0.05). The proteins from these spots were digested in gel and identified using mass spectrometry (MALDI TOF-TOF MS). In total, 31 DEPs were identified using Mascot database searching (Figure 5, Table 1, and Supplemental Table S1).

### 2.5. Annotation and Functional Categorization of DEPs

**Figure 6:**
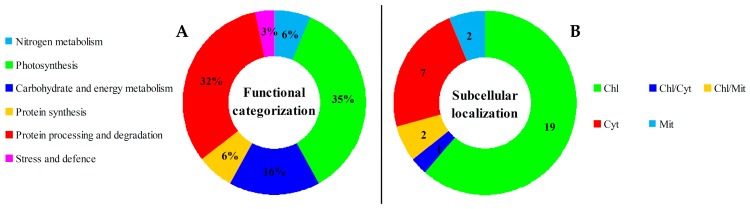
Functional categorization and subcellular localization of the differentially expressed proteins (DEPs) from various leaf regions at different development stages of *Hosta* “Gold Standard”. (**A**) A total of 31 DEPs were classified into six functional categories. The percentage of proteins in different functional categories is shown in the pie; (**B**) Subcellular localization categories of the identified proteins. The numbers of proteins with different locations are shown. Chl: Chloroplast; Cyt: Cytoplasm; Mit: Mitochondria.

Among the 31 identified DEPs, seven were originally annotated as unknown, or hypothetical proteins. In this study, they were re-annotated according to The Basic Local Alignment Search Tool (BLAST) analysis (Table 1, and Supplemental Table S2). Taken together, based on the function information from Gene Ontology, BLAST analysis, and literature, all these proteins were mainly involved in nitrogen metabolism, photosynthesis, carbohydrate and energy metabolism, protein synthesis, protein processing and degradation as well as stress and defense (Table 1). Among them, proteins involved in protein metabolism, including protein synthesis, protein processing and degradation, accounted for the largest group (38% of DEPs) (Figure 6A).

### 2.6. Proteoform Analysis of DEPs

The 31 DEPs only represented 21 unique proteins, since six proteins each had multiple proteoforms. These proteins were glutamine synthetase isoform GS1c (GS1) (spots 795 and 806), transketolase (TK) (spots 338 and 346), RuBisCO large subunit-binding protein subunit (RBP) (spots 459, 476, and 472), phosphoribulokinase (PRK) (spots 840 and 832), heat shock protein 70 (Hsp70) (spots 355, 361, 358, 367, and 332), and presequence protease 1 (PreP1) (spots 170 and 179) (Figure 5, Table 1, and Supplemental Table S3).

Interestingly, the proteoforms of each protein were distributed along a horizontal line on the 2DE gel (Figure 5), indicating that they have similar experimental molecular weight, but different isoelectric points (Table 1). Moreover, on the basis of our mass spectrometry (MS) identification, multiple alignments of the amino acid sequences from the six proteins were analyzed. The sequence alignment results indicate that there were high levels of sequence identities among various proteoforms of each protein (e.g., 100% of GS1 and PreP1, over 80% of TK, PRK, and Hsp70, as well as over 70% of RBP) (Supplemental Figure S2). Most importantly, the proteoform expression patterns of each protein were remarkably similar (Table 1, Supplemental Table S3). For example, two proteoforms of GS1 (spots 795 and 806) appeared at higher levels in MLM and MLC than that in YLM, YLC, and LENF. Besides, two proteoforms of PRK (spots 840 and 832) were increased in MLC and decreased in YLC. In addition, five proteoforms of Hsp70 (spots 355, 361, 358, 367, and 332) were all reduced in mature leaves when compared with young leaves, but kept stable level in LENF and YLM (Table 1, Supplemental Table S3).

### 2.7. Subcellular Localization of DEPs

**Figure 7:**
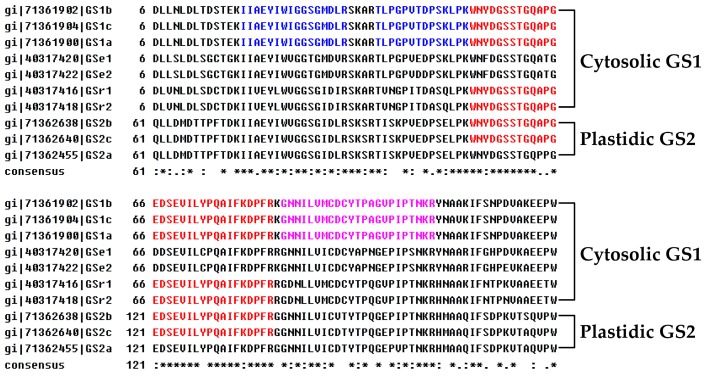
Sequence alignment between the mass spectrometry (MS)-identified peptides from two proteoforms of glutamine synthetase 1 (GS1) (spots 795 and 806) and the amino acid sequences of the ten GS isozymes in wheat (*Triticum aestivum* L.). The amino acid sequences from wheat used for the multiple sequence alignment were from National Center for Biotechnology Information non-redundant (NCBInr) protein database and submitted by Bernard *et al.* [36]. The accession numbers and names of the GS isozymes were listed on the left, and the subfamilies were shown on the right of the amino acid sequences. The asterisk indicates completely conserved residues; colon indicates highly conserved residues; dot indicates a weak conservative region. The MS-identified peptides from spots 795, 806 and both were highlighted in red, blue, and purple fonts, respectively. The results showed that the MS-identified peptides were well aligned with cytosolic GS1, but not plastidic GS2. The detailed information of the identified peptides can be found in Table S1, and the complete alignment can be found in Figure S3.

The analysis of protein subcellular localization is critical for understanding their functions. In this study, the 31 DEPs were submitted to five internet tools (*i.e.*, YLoc, LocTree3, Plant-mPLoc, ngLOC, and TargetP) for subcellular localization (Table 1, Figure 6B, and Supplemental Table S4). Among them, 22 DEPs (71%) were predicted to be localized in chloroplasts, including three DEPs localized in chloroplast and mitochondrion/cytoplasm (Figure 6B). The majority of DEPs were predicted to be localized in chloroplasts, suggesting that the leaf color changes were mainly attributed to the metabolic differences in the chloroplasts. In addition, our results of MS-identified peptides of DEPs provided amino acid sequence information for their subcellular localization. The sequence alignment of four proteins (GS, phosphoglycerate kinase (PGK), fructose-bisphosphate aldolase (FBA), and Hsp70) with MS-identified peptides and their homologs from other plant species (e.g., wheat, rice, and Arabidopsis) supported our prediction of subcellular localization. For example, the MS-identified peptides from two proteoforms of GS1 (spots 795 and 806) were submitted to alignment analysis with their homologs of ten GS family members in wheat [36]. The results indicate that they were well aligned with the cytosolic GS1, but not with the plastidic GS2 (Figure 7, Supplemental Figure S3). For PGK (spot 740) and FBA (spot 847), the MS-identified peptides of PGK were matched with chloroplast-localized homologs from rice (*Oryza sativa* sp. *japonica*) [37] (Supplemental Figure S4), while the MS-identified peptides of FBA were matched with four cytoplasm-localized FBA of eight family members from Arabidopsis [38] (Supplemental Figure S5). For Hsp70, the peptide sequences of five proteoforms were aligned with 14 homologs of Hsp70 in Arabidopsis [39]. The specific MS-identified peptides from four proteoforms (spots 355, 361, 358, and 367) were only matched to the cytoplasm-localized Hsp70s, while one proteoform (spot 332) was well aligned with the Hsp70 localized in the chloroplast (Table 1, Supplemental Figure S6).

### 2.8. Hierarchical Cluster Analysis of DEPs

**Figure 8:**
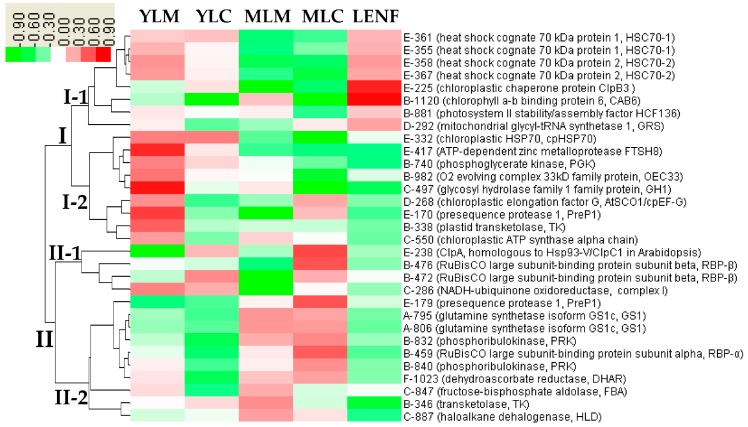
Hierarchical clustering analysis of the expression profiles of the identified 31 proteins. The five columns represent various leaf regions at different development stages of *Hosta* “Gold Standard”, including young leaf margin (YLM), young leaf center (YLC), mature leaf margin (MLM), mature leaf center (MLC), leaf under excess nitrogen fertilization (LENF). The rows represent individual proteins. Two main clusters (I and II) and subclusters of I (I-1 and I-2) and subclusters of II (II-1 and II-2) are shown on the left side. Functional categories indicated by capital letters, spot numbers, and protein names are listed on the right side. The scale bar indicates log (base2) transformed protein abundance ratios ranging from −0.9 to 0.9. The ratio was calculated by dividing percent volume (vol %) of each protein spot at various leaf region samples by the average vol % of the five various leaf region samples of the same protein spot. The increased and decreased proteins are represented in red and green, respectively. The color intensity increases with increasing abundant differences. Functional categories: A, nitrogen metabolism; B, photosynthesis; C, carbohydrate and energy metabolism; D, protein synthesis; E, protein processing and degradation; F, stress and defence.

To better understand the expression patterns of all the coordinately regulated proteins, hierarchical clustering analysis of the 31 DEPs were performed, which revealed two main clusters (Figure 8). Cluster I contained 17 DEPs, the abundances of which in mature leaves were reduced when compared with young leaves (especially in YLM). These proteins were grouped into two distinct subclusters. Subcluster I-1 included the significantly increased proteins in LENF when compared with mature leaves. The proteins in Subcluster I-1 mainly included four proteoforms of heat shock congnate 70 kDa (Hsc70) (spots 335, 358, 367, 361), and a chloroplastic chaperone protein ClpB3 (spot 225), as well as chlorophyll a/b binding protein 6 (CAB6) (spot 1120) and photosystem II stability/assembly factor HCF136 (HCF136) (spot 881). Subcluster I-2 contained the proteins whose abundances were induced in YLM compared with other tissues (e.g., PGK (spot 740) and chloroplastic elongation factor G (cpEF-G) (spot 268)) (Figure 8). Cluster II contained 14 DEPs, which were also grouped into two subclusters. Subcluster II-1 included the proteins that were increased in central regions but decreased in marginal regions of young leaves and mature leaves, as well as in LENF, such as the ClpA subunit (homologous to Hsp93-V/ClpC1 in Arabidopsis) (spot 238), two proteoforms of RBP-β (spots 476 and 472), and NADH-ubiquinone oxidoreductase (spot 286). Subcluster II-2 contained the proteins that were increased in mature leaves but decreased in young leaves and LENF, including two proteoforms of GS1 (spots 795 and 806), two proteoforms of PRK (spots 832 and 840), a proteoform of RBP-α (spot 459), dehydroascorbate reductase (DHAR) (spot 1023), FBA (spot 847), plastid TK (spot 338), and haloalkane dehalogenase (HLD) (spot 887) (Figure 8). These results suggest that the proteins appeared function skewed in different regions of variegated leaves of *Hosta* “Gold Standard”.

## 3. Discussion

### 3.1. Leaf Color Difference of Variegated Leaves Is Determined by the Chlorophyll Contents

It is well known that the leaf color is affected by chlorophyll and carotenoid levels in variegated leaves. In this study, the center color of variegated leaves was gradully turned from light green (in YLC) to golden (in MLC) during normal leaf development, and can be changed to green under excess nitrogen fertilization, but the margin color of variegated leaves has no obvious changes (Figure 1 and Figure 2). *Aurea* yellow leaves were severely deficient in all the pigments and particularly in Chl *a* and Chl *b* [20]. In *Hosta* “Gold Standard” leaves, total Chls, Chl *a*, and Chl *b* in golden regions were obviously lower than in green regions of variegated leaves, and the Chl *b* accumulation was more reduced than Chl *a* (Figure 3A–D). A similar phenomenon has also been observed in *Ligustrum vicaryi* [21] and *Amaranthus tricolor* [18]*. L. vicaryi* is a plant species with chlorophyll-less golden-color leaves on the upper canopy but green leaves under the canopy [21], and *A. tricolor* is a C_4_ plant with three-color (green, yellow and red) leaves [18]. After 30 days of excess nitrogen fertilization, the average total Chls, Chl *a* and Chl *b* in LENF were increased obviously when compared with that in central regions, but still lower than that in marginal regions of young leaves and mature leaves (Figure 3A–C). The high abundant Chls in marginal regions ensured higher levels of photosynthesis and other metabolisms, which might compensate for the lower levels in central regions of variegated leaves [17,40].

### 3.2. Nitrogen Metabolism Level Determined the Chlorophyll Synthesis in Variegated Leaves

**Figure 9:**
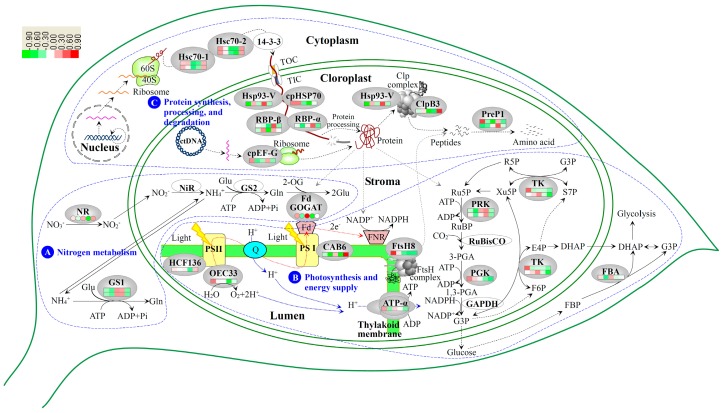
Schematic presentation of the leaf color regulation mechanism in chimera *Hosta* “Gold Standard” leaves. The identified proteins were integrated into subcellular pathways. A, Nitrogen metabolism; B, Photosynthesis and energy supply; C, Protein synthesis, processing and degradation. Protein expression patterns and enzyme activities are marked in the oval shapes with squares and circles in different colors, respectively. The increased and decreased proteins are represented in red and green, respectively. The color intensity increases with increasing abundant differences. The solid line indicates single-step reaction, and the dashed line indicates multistep reaction. Abbreviation: 2-OG, 2-oxoglutarate; 40S, eukaryotic small ribosomal subunit; 60S, eukaryotic large ribosomal subunit; ATP-α, ATP synthase alpha chain; CAB6, chlorophyll a/b binding protein 6; ClpB3, chloroplastic chaperone protein ClpB3; cpEF-G, chloroplastic elongation factor G; cpHsp70, stromal 70 kDa heat shock-related protein; DHAP, dihydroxyacetone phosphate; E4P, erythrose-4-phosphate; F6P, fructose 6-phosphate; FBA, fructose-1,6- bisphosphate aldolase; FBP, fructose 1,6-bisphosphate; Fd, ferredoxin; FNR, ferredoxin-NADP reductase; FtsH8, ATP-dependent zinc metalloprotease FtsH8; G3P, glycerate 3-phosphate; GAPDH, Glyceraldehyde-3-phosphate dehydrogenase; Gln, glutamine; Glu, glutamate; GS1, cytosol-localized glutamine synthetase isoform; GS2, plastid-localized glutamine synthetase isoform; HCF136, photosystem II stability/assembly factor HCF136; Hsc70, heat shock congnate 70 kDa protein; Hsp93-V, heat shock protein 93-V(ClpC1, ClpA subunit); NiR, nitrite reductase; NR, nitrate reductase; OEC33, O_2_ evolving complex 33kD family protein; PGA, 3-phosphoglyceric acid; PGK, phosphoglycerate kinase; PreP1, presequence protease 1; PRK, phosphoribulokinase; PSI, photosystem I; PSII, photosystem II; Q, quinine; R5P, ribose-5-phosphate; RBP, RuBisCO large subunit-binding protein subunit; Ru5P, ribulose-5-phosphate; RuBisCO, ribulose-1,5-bisphosphate carboxylase; RuBP, ribulose-1,5-bisphosphate; S7P, sedoheptulose-7-phosphate; TIC, translocon at the inner envelope membrane of chloroplasts; TK, transketolase; TOC, translocon at the outer envelope membrane of chloroplasts; Xu5P, xylulose-5-phosphate.

Nitrogen nutrition (*i.e.*, nitrate and ammonium) is one of the major determinants of chlorophyll synthesis and chloroplast development in variegated leaves [41]. About 75% of the total nitrogen in plant is required for chlorophyll synthesis, the components of photosynthetic enzymes and thylakoid membranes (reviewed in [41]). Nitrate needs to be reduced to nitrite by NR in roots, and then be reduced to ammonium by nitrite reductase (NiR) in chloroplast. Subsequently, ammonium from various sources (e.g., soil, nitrate reduction, photorespiration, and amino acid decomposition) is fixed into amino acids (*i.e.*, glutamine/glutamate) in the GS/GOGAT pathway [42]. GS catalyzes the ATP-dependent condensation of ammonium to the δ-carboxyl group of glutamate (Glu) to form glutamine (Gln), while GOGAT catalyzes the conversion of Gln and 2-oxoglutarate to two molecules of Glu, which serves as one of the nitrogen donors for the biosynthesis of organic nitrogenous compounds, such as chlorophylls, proteins, and nucleotides [43] (Figure 9). Therefore, NR, GS, and GOGAT are the key enzymes in nitrogen assimilation processes, which play important roles in chlorophyll synthesis and chloroplast development.

In *Hosta* “Gold Standard” leaves, obviously lower levels of nitrogen metabolism in center light green/golden regions than in margin regions of young and mature variegated leaves could be inferred from the results of total nitrogen levels and activities of NR and GOGAT (Figure 4A–C). However, the activities of GS and the levels of two proteoforms of GS1 showed exactly opposite patterns in mature leaves compared to young leaves (Figure 4D, Table 1, Figure 9). In most C_3_ plant leaves, GS family includes cytosol-localized GS1 and chloroplast-localized GS2. GS activity in leaves is mainly attributed to GS2 (70%–95%), but not GS1 (5%–30%) [44]. GS1 mainly functions in nitrogen remobilization from protein breakdown in leaves, especially the remobilization of the inorganic form of nitrogen from senescing leaves for grain filling [45]. GS2 mainly functions in the reassimilation of ammonia generated from photorespiration [45] and nitrate reduction [46]. Therefore, although GS1 levels were increased in mature variegated leaves of C3 plant *Hosta*, the decrease of total GS activity in mature leaves was probably due to the decline of GS2 activity (Figure 4D). It has been found that the decreases of GS2 abundance occurred in an albinism line of winter wheat [7] and at the albinistic stage in an albino tea cultivar [8]. This indicates that reassimilation of ammonia released from photorespiration was reduced in mature variegated leaves. This is supported by the decreases of photorespiration and photosynthesis rates due to the decreased abundances of photosynthetic proteins (Table 1). Moreover, the induced abundance of GS1 in mature variegated leaves implied that assimilation of ammonium from the degraded proteins is critical for variegated leave maturation. Interestingly, in leaves during senescence [47], under salt stress [48], or pathogen attack [49], GS activity also decreased, accompanied by the induced GS1 abundance and reduced GS2 abundance.

In addition, nitrogen metabolism was inhibited in central light green/golden regions in variegated leaves, and recovered after excess nitrogen fertilization (Figure 4B–D). Similar nitrogen metabolic processes have been found in chloroplast-ribosome-deficient leaves of “*albostrians*” mutant of barley and “*iojap*” mutant of maize [50]. In these albino mutants, the NR activity was generally 40% lower than wide-type plants [50]. In addition, proteomic analysis has revealed that GS abundance was decreased in albinistic stage, and then increased in the regreening stage in the albino tea cultivar [8]. Among the three enzymes, GS activities in LENF were induced, but GS1 abundances in LENF were lower than those in mature leaves. This indicates that the induced GS activity in LENF was due to the increase of GS2 activity, because it has been shown that excess ammonia can induce GS2 gene transcription in both rice and tobacco leaves [51]. The chloroplast-localized GS2 contributed more Gln for plastid development [52]. Inhibition of GS with the herbicide phosphinothricin (PPT) led to decreases of GS2 mRNA and polypeptides, but increase of GS1, and ultimately rapid chlorosis of leaves [46]. The GS1 subfamily has several members in some plants (e.g., maize [53], Arabidopsis [54], and rice [55]), with different expression patterns and various affinities for NH_4_^+^ (reviewed in [45]). The alignment analysis indicates that two proteoforms of GS1 in our results were homologous with GS1a/GS1b/ GS1c of wheat and GS1-3/GS1-4 of maize [36]. The two proteoforms of GS1 were consistently expressed in young leaves and LENF, but obviously increased in mature leaves (Table 1, Figure 9). Their homologs in maize seedlings have no obvious changes after external ammonium application [53]. In addition, the two proteoforms of GS1 with similar molecular weight may be generated from post-translational modifications (e.g., phosphorylation), since GS1 activity is regulated by phosphorylation and interaction with 14-3-3 protein during senescence in relation to nitrogen remobilization [47]. All these data indicate that assimilation patterns of ammonium coordinately catalyzed by GS1 and GS2 is critical for many processes of development and stress response (Figure 9).

### 3.3. Chloroplast Development in Different Stages and Nitrogen Levels

In the leaves of many variegated plant species, chloroplast development is different in various regions, nitrogen levels, light intensities, and other environmental conditions [19,21,22]. Leaves under sufficient nitrogen conditions have large chloroplasts with well-developed grana, stroma lamellae, thylakoid membranes, and photosynthetic enzymes, and starch granules [41,56]. Several PS related proteins (e.g., CAB6, HCF136, and O_2_ evolving complex 33 kD family protein (OEC33)) identified in this study are critical for thylakoid membrane structure construction and photosynthesis regulation in variegated leaves. CAB6, HCF136, and OEC33 were decreased in light green/golden regions in young and mature leaves, and induced after excess nitrogen fertilization (Table 1, Figure 9). CAB6 is a constituent of PSI antenna encoded by *LHCa1* gene, and was found to be reduced in the albino leaves compared to the green leaves from bamboo (*Pseudosasa japonica*) [57]. CAB was also increased back to the normal level during leaf recovery from senescence to green leaves [58]. HCF136 is a stroma thylakoid lumenal side-localized PSII assembly factor required for PSII proteins accumulation [59]. The severe mutational defect of HCF136 led to a pale green seedling phenotype [59]. OEC also bounds to the lumen side of PSII, and functions as a water-oxidizing enzyme in the water photooxidation process [60]. It has been reported that some LHCII proteins were deficient in the yellow-leaved mutant of *H. sieboldii* (“Wogan Gold”) [22] and maple (*Acer negundo* Hassk. var. *odessanum*) [23], or appeared in low levels in golden leaves from *L. vicaryi* [21]. Importantly, the expression pattern of PS proteins was consistent with our previous observation of chloroplast ultrastructure of *Hosta* “Gold Standard”, which proved that thylakoid membrane and grana formation in the golden region was obviously affected, but normal in green region and green leaves after excess nitrogen fertilization [19]. Previous findings in another *Hosta* species (*Hosta sieboldii*) and *L. vicaryi* reported that the grana formation and chloroplast development were blocked in the leaves of yellow mutant, and leaves contain only a few separate thylakoid membranes, and the grana are degraded [22], as well as the chloroplast ultrastructure was different in upper golden leaves, lower green leaves, and regreening upper leaves under different light intensities [21].

### 3.4. Photosynthesis and Energy Supply Are Different in Various Regions of Variegated Leaves

It is well-known that photosynthesis capacity in the yellow/golden regions is generally lower than in the green regions of variegated leaves of several species, such as willow myrtle (*Agonis flexuosa* (Willd.) Sweet), oleander (*Nerium oleander* L.) [16], agave (*Agave americana*) [17], and fig tree (*Ficus microcarpa* L. f. cv Golden Leaves) [13], but the molecular mechanisms underlying the photosynthetic differences are unknown. In this study, besides the three photosystem proteins (CAB6, HCF136, OEC33), we identified five enzymes related to the Calvin cycle, which were RBP-α, RBP-β, PGK, TK, and PRK (Table 1, Figure 9). The decreases of CAB6, HCF136, and OEC33 in golden regions of variegated leaves would lead to low light reaction activity and shortage of ATP and NADPH for Calvin cycle. Accordingly, PGK and one TK proteoform also appeared to be low in the center golden regions, suggesting the reduced photosynthetic carbon reduction in the golden regions of variegated leaves. In contrast, RBPs and PRKs were induced in the center golden regions of variegated leaves, appearing contradictory to the reduced photosynthesis in the golden regions. Although RBP is normally considered as chloroplast chaperonin 60 (Cpn60), which interacts with RuBisCO for positive regulation of its function in photosynthetic tissues [61], they also can bind to other imported proteins (e.g., stromal plastid division protein and FtsZ polymer), regulating protein folding and dynamics for the formation of normal plastid division apparatus [62,63]. Taken together with the unchanged abundance of RuBisCO in our results, we speculate that the induced RBPs (Cpn60) mainly contribute to other imported protein folding in variegated leaves of *Hosta* “Gold Standard”. Additionally, PRK is an essential enzyme in the Calvin cycle for regeneration of the RuBisCO substrate [64], and it was also reported to have negligible control of the Calvin cycle under a range of environmental conditions [65]. The photosynthetic carbon assimilation in golden regions of variegated leaves would be controlled by the rate-limiting enzyme TKs, but not PRKs.

After excess nitrogen fertilization, the levels of these Calvin cycle-related enzymes were decreased, although the photosynthesis system proteins (CAB6, OEC33, and HCF136) were increased in LENF when compared with those in MLC (Table 1, Figure 9). The decreased photosynthesis was also found in *Hosta* “Blue Umbrella” [66] and *Hosta* “Francee” [67] when fertilized with more than 0.5 g/kg urea. All these results indicated that nitrogen level was strongly correlated with photosynthetic capacity [68,69]. Nitrogen assimilation into Glu in leaves is dependent on the consumption of carbon skeletons and supply of ATP and NADPH generated from photosynthesis [70]. In our results, the increased nitrogen assimilation would compete against ATP/NADPH with photosynthetic carbon fixation, leading to the carbon assimilation decrease in LENF [71]. In addition, the difference of photosynthetic CO_2_ assimilation would lead to the carbohydrate metabolism changes in various regions of variegated leaves. In the variegated leaves from *A. americana*, the contents of various sugars (e.g., sucrose, glucose, and fructose) were lower in the yellow regions than in the green regions [17]. In this study, we found that the abundance of cytoplasm-located FBA was lower in the central regions than in the marginal regions, indicating glycolysis and gluconeogenesis would be reduced in the golden regions of variegated leaves (Table 1, Figure 9).

### 3.5. Chloroplast Protein Synthesis, Processing and Degradtaion in Variegated Leaves

Protein synthesis and processing in chloroplasts is crucial for chloroplast biogenesis, development and metabolism [72]. It has been reported that ribosome numbers are much reduced in *aurea* yellow leaves [20]. In this study, we found an induced cpEF-G in golden regions of mature leaves (Table 1, Figure 9), which is encoded by *cpEF-G* in plastid genome. Its homolog in Arabidopsis is snowy cotyledon1 (SCO1). A *sco1-1* mutant exhibited greening inhibition during the early seedling development stage, while the *sco1* null mutant cannot survive [73]. The induced SCO1 functions in the increase of protein translation to compensate for the reduced amount of ribosome in a *clpR4* mutant [74]. Thus, the increased cpEF-G would contribute for the stabilization of protein translation in the golden regions of variegated leaves.

The polypeptide maturation and protein quality control are important for proper plastid differentiation and chloroplast development [75]. It has been found that chaperones and proteases, such as cpHsp70 [76,77], ClpB3 [78], caseinolytic protease, subunit C (ClpC) [79], FtsH8 [80], and PreP1 [81] were involved in leaf variegation. The Arabidopsis mutants of these genes shared some similar characteristics with the variegated leaves of *Hosta* “Gold Standard”, such as leaf variegation, decreased pigment content, and reduced chloroplast development [76,77,78,79,80,81]. In our results, the abundances of ClpB3, Hsp93-V (ClpC1), PreP1, and FtsH8 showed obvious changes in various regions of variegated leaves (Table 1, Figure 9). ClpB3 is a chloroplast-localized chaperone. It functions in the unfolding of aggregated protein cooperating with the DnaK chaperone that is homologous to cpHsp70 [82]. The *ClpB3* mutants (e.g., *albino* or *pale-green*) showed severe defects in chloroplast development [78]. The low abundance of ClpB3 in the variegated leaves of *Hosta* “Gold Standard” indicates that it would contribute to the reduced protein solubility and/or assembly processes in the stroma [79]. Besides, soluble stroma-localized Hsp93 (ClpC) is also thought to be a chaperone component of the Clp protease complexes, which plays a central role in dissolution and degradation of protein aggregates as a stromal housekeeping protease [83,84]. Interestingly, in Arabidopsis, it was also shown that the Hsp93-V (ClpC1) participates in the degradation of chlorophyllide a oxygenase (CAO), which is responsible for the conversion of Chl *a* to Chl *b* [85,86]. Therefore, the increased Hsp93-V (ClpC1) in the golden region of variegated leaves of *Hosta* “Gold Standard” might induce the degradation of CAO, leading to the reduction of conversion of Chl *a* to Chl *b*. This would be the reason of increased ratio of Chl *a*/*b* in the golden regions of the leaves (Figure 3D). In addition, PreP1 is a zinc-dependent metalloendopeptidase. It functions as the organellar peptide degrading protease responsible for degrading free targeting peptides and clearing other unstructured toxic peptides [87]. The protease activity has been shown to be increased in chlorophyll-less leaves, such as albino leaves [8,88] and senescent leaves [89], indicating they may hydrolyze chlorophyll-binding proteins during Chl degradation [58]. The similar trends of ClpB3 and cpHsp70 in this study confirmed the synergistic functions of these chaperones to unfold aggregated proteins followed by protein refolding [90]. Therefore, in our study, the reduced cpHsp70 and ClpB3 might lead to accumulation of unwanted/damaged proteins in the golden regions of variegated leaves especially, and the increased Hsp93-V (ClpC1) and PreP1 might contribute to degrading these aggregates in the golden regions. Additionally, the chloroplast FtsH complex is in charge of the turnover of the Photosystem II reaction center D1 protein, as well as other protein degradation for the development and maintenance of the photosynthetic apparatus [91]. Early chloroplast development requires sufficient FtsH for thylakoid biogenesis [75]. The *ftsh2 ftsh8* double mutants exhibiting an albino-like phenotype emphasize the possibility that both FtsH2 and FtsH8 are necessary for the proper FtsH function [80]. Leaf variegation is suppressed, at least in part, by an increase in FtsH levels [74]. In this study, FtsH8 appeared at lower levels in the variegated mature leaves than in young leaves, suggesting that thylakoid formation was affected in the variegated leaves of *Hosta* “Gold Standard”. After excess nitrogen fertilization, the nitrogen assimilation, amino acid metabolism, and protein synthesis are enhanced in the leaves. The increased Hsc70s, cpHsp70 and ClpB3 in LENF would contribute to folding of nascent polypeptide chains, prevention of aggregation, and solubilization/refolding of aggregated proteins [92]. In addition, the decline of Hsp93-V (ClpC1) and PreP1 in LENF indicates that protein degradation was inactive after excess nitrogen fertilization. Similar decreases in protease activities have also been reported in the regreening leaves compared with senescent leaves [58] and albescent leaves [88].

Importantly, protein import from cytosol to developing chloroplasts through translocons at the outer and inner membrane of chloroplasts is necessary for chloroplast differentiation and development [75]. Various cytosolic chaperones (e.g., Hsp70 and Hsp90) are considered to interact with unfolded preproteins for maintaining their import competence and directing them to the translocons [93]. More than 75% of chloroplast transit peptides are predicted to have at least one Hsp70 binding site [94]. cpHsp70, a component of the protein import machinery of the chloroplasts, is crucial for the import of nuclear-encoded polypeptides (e.g., the light harvesting chlorophyll a/b-binding protein (prLHCBP)) [95,96] (Figure 9). When two stromal cpHsc70-1 and cpHsc70-2 were knocked out in Arabidopsis, protein import into the chloroplasts was affected in the early developmental stages of mutants [76]. A T-DNA insertion line of *cpHsc70-1* (*ΔcpHsc70-1*) has variegated cotyledons [77]. In this study, four cytoplasm-located and one chloroplast-located proteoform of Hsp70 appeared at low abundances in variegated leaves of *Hosta* “Gold Standard” (Table 1, Figure 9). Hsp70 proteoforms were also reduced in leaves at albinism stage of winter wheat [7] and the albinistic stage of an albino tea cultivar “White leaf No. 1” [8]. The decrease of Hsp70s in chlorophyll-less leaves may affect chloroplast protein import, leading to non-green leaf color [7,8]. However, we also found Hsp93-V (ClpC1) and RBPs (Cpn60s) have significantly higher abundance in golden leaves, but lower abundance in LENF. RBPs (Cpn60s) contribute to the imported protein to fold into native conformation by interacting with a component of the inner membrane import apparatus Tic110 [97]. The expression patterns of Hsp93-V (ClpC1) and cpHsp70 in this study proved the previous notion that cpHsp70-1 and Hsp93-V (ClpC1) have redundant functions in chloroplast protein import [76].

## 4. Materials and Methods

### 4.1. Materials and Treatment

*Hosta* “Gold Standard” grew at Beijing Botanical Garden (39°9′N; 116°4′E), Institute of Botany, Chinese Academy of Science. Uniformly-sized field three-year-old seedlings of *Hosta* “Gold Standard” were selected and transplanted to flowerpots (28 cm in diameter, 22 cm in height). Each flowerpot contained one seedling and 5 kg of soils, including garden soil, sand, and humus (2:1:1, *v*/*v*/*v*). And its basic contents were as follows: total nitrogen, 0.22%; soluble phosphorus, 0.127%; total kalium, 2.38%; organic matter, 4.36%; and pH 7.403. Owing to reduced biomass accumulation over 50% shadings of the *Hosta* plants [98], 50% of natural light was selected in this study.

Uniformly-sized potted seedlings were then placed in the shade with 50% of natural light by using black shade in mid-April, when YLM and YLC were harvested. About 70 days later, MLM and MLC were harvested from the variegated mature leaves. After this harvest, excess nitrogen fertilizer (2 gram urea per kilogram soil, 46%) was applied for the remaining variegated mature leafed plants according to the methods from Liu *et al.* [19]. After 30 days treatment, LEFN were harvested. All these five samples were used freshly, or immediately frozen in liquid nitrogen and then stored at −80 °C for further experiments.

### 4.2. Leaf Color Quantification

The leaf color was quantified by using a color meter (NF333 spectrophotometer, Nippon Denshoku Industries Co., Tokyo, Japan). Results were expressed according to CIELAB color coordinates system, L*, a*, and b*. L* represents the perceived lightness, and color coordinate a* and b* indicate the change in hue from red to green and from yellow to blue, respectively [99].

### 4.3. Chlorophyll Content Determination

The contents of Chl *a*, Chl *b* and total Chls were determined according to the method described by Arnon [100]. Fresh 6 mm diameter leaf discs were homogenized with 80% (*w*/*v*) cold acetone (Jiangsu Qiangsheng Chemical Co., Ltd., Jiangsu, China), followed by centrifuged at 5000× *g* for 10 min. The chlorophyll contents were calculated from the absorbance of the supernatant at 663 and 646 nm according to the method of Lichtenthaler and Wellburn [101].

### 4.4. Leaf Nitrogen Content Determination

Fresh leaves were cleaned, dried, smashed, screened through mesh size of 80, and digested with sulfuric acid-hydrogen peroxide. Then the total nitrogen content was determined by using a Kjeltec 2300 Auto analyzer (Foss Tecator AB, Höganas, Sweden).

### 4.5. Nitrogen Metabolism-Related Enzymes Activity Assay

The activities of NR and GS were determined according to Zhao *et al.* [102]. The activity of GOGAT was determined by the method of Singh and Srivastava [103] with some modification. The extraction buffer contained 1 mM EDTA (Amresco, Solon, OH, USA), 1 mM β-mercaptoethanol (Amresco, Solon, OH, USA), 1 mM MgCl_2_ (Richjoint Chemical, Shanghai, China), 10 mM Tris (Amresco, Solon, OH, USA)-HCl (pH 8.2). The assay buffer contained 0.4 mL 20 mM l-glutamine (Sigma-Aldrich Co., St. Louis, MO, USA), 0.5 mL 20 mM α-oxoglutarate (Sigma-Aldrich Co., St. Louis, MO, USA), 0.1 mL 10 mM KCI (Richjoint Chemical, Shanghai, China), 0.2 mL 3 mM NADH (F. Hoffmann-La Roche Ltd., Basel, Switzerland) and 0.3 mL of the enzyme preparation. The final volume was completed to 3.0 mL with 25 mM Tris-HCl buffer (pH 7.6) [103]. The reaction was started by the addition of l-glutamine immediately following the enzyme preparation. The absorbance decrease was recorded for 4 min at 340 nm by using a UV-1800 spectrophotometer (Shimadz, Kyoto, Japan). The activity of GOGAT was expressed as the amount of NADH oxidized per minute per milligram protein.

### 4.6. Protein Sample Preparation, 2-DE, and Image Analysis

The proteins from leaves were extracted using a phenol extraction method described in detail in Wang *et al.* [104]. The experiments were repeated three times using protein samples prepared independently from different batches of plants. Protein concentration was determined using a Quant-kit according to the manufacture’s instructions (GE Healthcare Life Science, Uppsala, Sweden). Protein samples were separated using 24 cm, pH 4–7 linear gradient IPG strips through isoelectric focusing (IEF) in the first dimension, followed by 12.5% SDS-PAGE gels in the second dimension [105]. Gel images were acquired by scanning the CBB-stained gel using an ImageScanner III (GE Healthcare Life Science, Uppsala, Sweden) at a resolution of 300 dpi and 16-bit grayscale pixel depth. Then, the images were analyzed using ImageMaster 2D software (version 5.0) (GE Healthcare Life Science, Uppsala, Sweden). For quantitative analysis, the volume of each spot was normalized against the total valid spots. Spots from three biological replicates with more than a 1.5-fold change among the protein samples and a *p* value smaller than 0.05 were considered to be DEPs.

### 4.7. Protein Identification and Database Searching 

The differentially expressed spots were cut from the gels and digested with trypsin according to a previous method [105]. Proteins were identified according to the method described in Sun *et al.* [106]. The MS and MS/MS spectra acquired on a MALDI TOF-TOF mass spectrometer (4800 Proteomics Analyzer, AB SCIEX, Foster City, CA, USA) were searched against the National Center for Biotechnology Information non-redundant (NCBInr) protein databases [107] (10,348,164 sequences entries in NCBInr) using the search engine Mascot (Matrix Sciences, London, UK) [108]. The taxonomic category was Green Plants (730,150 sequences). The searching parameters were set according to Wang *et al.* [104], including mass accuracy of 0.3 Da, one missed cleavage per peptide allowed, carbamidomethylation of cysteine as a fixed modification, and oxidation of methionine as a variable modification. To obtain high confident identification, proteins had to meet the following criteria: the top hits on the database searching report, the MOWSE score greater than 43 (*p* < 0.05), and at least two peptides matched with nearly complete y-ion series and complementary b-ion series present.

### 4.8. Protein Classification

The identified proteins were searched against the NCBI database [107] and UniProt database [109] to determine if their functions were known. Based on the function information from Gene Ontology, BLAST alignments, and literature, these proteins were grouped into various categories.

### 4.9. Protein Subcellular Location

The subcellular locations of the identified proteins was predicted using five internet tools according to Zhao *et al.* [110] with some modifications: YLoc [111], confidence score ≥0.7; LocTree3 [112], expected accuracy ≥80%; Plant-mPLoc [113], no threshold value in Plant-mPLoc; ngLOC [114], probability ≥60%; and TargetP [115], reliabilityclass ≤4. Only the consistent predictions from at least two tools were accepted as a confident result.

### 4.10. Multiple Sequence Alignment

Multiple sequence alignments of related proteins were created by using ClustalX 1.81, and refined by using BoxShade Server [116].

### 4.11. Hierarchical Cluster Analysis

Self-organizing tree algorithm hierarchical clustering of the protein expression profiles was performed using Cluster software (version 3.0) as described in the website [117]. Input data was preprocessed by dividing percent volume (vol %) of each protein spot at various leaf region samples by the average vol % of the five various leaf region samples of the same protein spot, and then followed by a log (base 2) transformation.

### 4.12. Statistical Analysis

All results were presented as means ± standard error (SE) of at least three replicates. Data were analyzed by One-Way Analysis of Variance (ANOVA) using the statistical software SPSS 17.0 (SPSS Inc., Chicago, IL, USA). A *p* value less than 0.05 was considered statistically significant [118].

## 5. Conclusions

High-throughput proteomics has shown advantages in acquiring large-scale information on individual proteins and their dynamic changes underlying sophisticated leaf color-related cellular processes. We discovered 31 proteins related to the leaf color regulation in *Hosta* “Gold Standard”, represented by the induced GS1 and the decreased Hsp70 in mature variegated leaves, as well as the highly abundant cpEF-G and RBP (Cpn60) in the center golden regions of variegated leaves compared to the green regions of variegated leaves. These protein expression patterns implied low levels of nitrogen metabolism, photosynthesis, and energy supply, dissolution and degradation of those unwanted/damaged proteins aggregates in the golden regions, as well as inhibition of protein import from cytosol to developing chloroplasts in the mature variegated leaves. Additionally, diverse proteoforms of several proteins (e.g., GS1, Hsp70, RBP, PRK, and PreP1) implied post-translational modification performs key functions during the development of variegated leaves. All these findings provide useful molecular information for better understanding the complicated leaf color regulation mechanisms. However, further validation and characterization of the proteins identified in this study are needed. Moreover, investigation of low abundant and extremely acidic/basic proteins in the varigated leaves, as well as analysis of the protein post-translational modification and protein-protein interaction using proteomics approaches are also necessary for further understanding of the complex cellular and molecular processes in variegated leaves.

## Figures and Tables

**Table 1:** Identified differentially expressed proteins of various leaf regions at different development stages of *Hosta* “Gold Standard”.

Spot No. *^a^*	Protein Name *^b^*	SCL *^c^*	Plant Species *^d^*	gi Number *^e^*	Exp. *M*w (Da)/pI *^f^*	Thr. *M*w (Da)/pI *^g^*	Sco *^h^*	Cov (%) *^i^*	QM *^j^*	V% ± SE *^k^* YLM MLM LENF YLC MLC
Nitrogen metabolism (2)										
795	glutamine synthetase isoform GS1c (GS1)	Cyt	*Triticum aestivum*	71361904	39,676/5.58	39,445/5.41	89	15	2	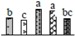
806	glutamine synthetase isoform GS1c (GS1)	Cyt	*T. aestivum*	71361904	39,505/5.75	39,445/5.41	168	15	3	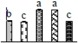
Photosynthesis (11)										
1120	chlorophyll a/b-binding protein type I, chlorophyll a-b binding protein 6 (CAB6) *	Chl	*Malus x domestica*	33772151	23,327/5.86	15,693/5.05	101	19	3	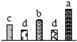
881	photosystem II stability/assembly factor HCF136, chloroplastic	Chl	*Vitis vinifera*	225423755	35,844/5.32	44,473/6.92	575	37	12	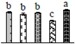
982	O_2_ evolving complex 33kD family protein (OEC33)	Chl	*Populus trichocarpa*	224084209	28,767/5.00	35,348/5.89	490	56	12	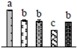
740	phosphoglycerate kinase (PGK)	Chl	*Ricinus communis*	255544584	41,980/5.63	50,114/8.74	597	35	11	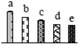
338	plastid transketolase (TK)	Chl	*Nicotiana tabacum*	194396261	78,879/5.95	80,451/6.16	141	17	10	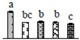
346	transketolase (TK)	Chl	*P. trichocarpa*	224063766	79,837/5.87	81,122/5.97	337	13	9	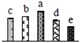
459	hypothetical protein SORBIDRAFT_09g014430, RuBisCO large subunit-binding protein subunit alpha (RBP-α) *	Chl	*Sorghum bicolor*	242090109	66,151/4.84	60,914/5.07	233	31	13	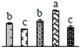
476	RuBisCO large subunit-binding protein subunit beta (RBP-β)	Chl	*V. vinifera*	225442531	63,140/5.18	65,255/5.62	474	17	8	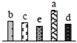
472	function unknown, RuBisCO large subunit-binding protein subunit beta (RBP-β) *	Chl	*Zea mays*	223948025	64,795/5.24	61,969/5.42	524	30	11	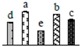
840	phosphoribulokinase (PRK)	Chl	*Pisum sativum*	1885326	38,189/4.99	39,230/5.41	113	13	3	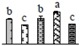
832	function unknown, chloroplastic phosphoribulokinase (PRK) *	Chl	*Glycine max*	255646270	38,858/4.85	45,757/5.89	277	38	11	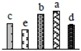
Carbohydrate and energy metabolism (5)										
847	fructose-bisphosphate aldolase (FBA)	Cyt	*Pandanus amaryllifolius*	40716077	37,892/6.55	38,642/6.48	236	26	6	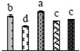
497	glycosyl hydrolase family 1 family protein (GH1)	Chl	*P. trichocarpa*	224135485	61,209/5.61	59,060/9.16	79	24	9	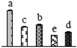
286	NADH-ubiquinone oxidoreductase (complex I)	Mit	*R. communis*	255582280	86,431/5.81	81,687/6.56	630	21	13	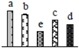
550	chloroplast ATP synthase alpha chain	Chl	*Yucca schidigera*	69214356	55,476/5.31	55,406/5.35	276	44	18	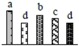
887	haloalkane dehalogenase (HLD)	Chl	*V. vinifera*	225442892	35,349/5.18	42,368/6.17	99	9	2	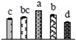
Protein synthesis (2)										
268	function unknown, chloroplastic elongation factor G (cpEF-G) *	Chl	*Arabidopsis thaliana*	14532624	90,396/5.21	86,287/5.48	585	25	15	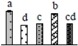
292	mitochondrial glycyl-tRNA synthetase 1 (GRS)	Mit	*V. vinifera*	225456501	86,133/6.16	79,370/5.98	108	5	3	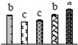
Protein processing and degradation (10)										
355	heat shock cognate 70 kDa protein 1 (Hsc70-1)	Cyt	*V. vinifera*	225449497	77,129/5.15	71,525/5.17	1050	61	26	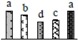
361	heat shock cognate 70 kDa protein 1 (Hsc70-1)	Cyt	*V. vinifera*	225449497	77,663/5.10	71,525/5.17	959	58	25	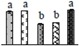
358	heat shock protein, heat shock cognate 70 kDa protein 2 * (Hsc70-2)	Cyt	*R. communis*	255573627	78,743/5.06	71,276/5.14	977	53	23	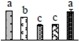
367	heat shock cognate 70 kDa protein 2 (Hsc70-2)	Cyt	*V. vinifera*	225434984	76,864/5.20	71,589/5.17	847	57	25	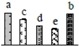
332	hypothetical protein VITISV_006707, stromal 70 kDa heat shock-related protein (cpHsp70) *	Chl	*V. vinifera*	147805297	80,390/4.75	69,198/5.11	722	36	16	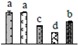
225	hypothetical protein VITISV_009951, chloroplastic chaperone protein ClpB3 *	Chl	*V. vinifera*	147842424	102,884/5.89	89,292/5.28	324	28	16	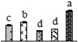
238	ATP-dependent Clp protease ATP-binding subunit clpA homolog CD4B (ClpA)	Chl	*Solanum lycopersicum*	399213	98,201/5.59	102,463/5.86	719	34	27	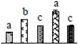
170	zinc metalloprotease (insulinase family), homologous to presequence protease 1 (PreP1) *	Chl/Mit	*A. thaliana*	9294618	113,711/4.99	118,265/5.32	346	13	11	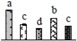
179	zinc metalloprotease (insulinase family), homologous to presequence protease 1 (PreP1) *	Chl/Mit	*A. thaliana*	9294618	113,515/5.07	118,265/5.32	157	11	9	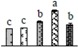
417	AC007592_12 F12K11.22, ATP-dependent zinc metalloprotease FtsH8 *	Chl	*A. thaliana*	6692685	71,862/5.35	71,014/5.81	144	13	5	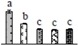
Stress and defence (1)										
1023	dehydroascorbate reductase (DHAR) class glutathione transferase DHAR1	Chl/Cyt	*P. trichocarpa*	283135904	27,322/6.05	24,525/4.93	143	10	2	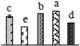

*^a^*: Assigned spot number as indicated in Figure 5; *^b^*: The name and functional categories of the proteins identified by MALDI TOF-TOF MS. Protein names marked with an asterisk (*) have been edited based on The Basic Local Alignment Search Tool (BLAST) against National Center for Biotechnology Information non-redundant (NCBInr) protein database. The detailed information of the NCBI BLAST can be found in Table S2; *^c^*: Protein subcellular localization predicted by software YLoc, LocTree3, Plant-mPLoc, ngLOC, and TargetP. SCL, subcellular location; Chl, chloroplast; Cyt, cytoplasm; Mit, mitochondria; *^d^*: The plant species that the peptides matched to; *^e^*: Database accession numbers from NCBInr; *^f^*^,*g*^: Experimental (f) and theoretical (g) molecular weight (Da) and pI of identified proteins. Experimental values were calculated using Image Master 2D Platinum Software. Theoretical values were retrieved from the protein database; *^h^*: The Mascot score obtained after searching against the NCBInr database; *^i^*: The amino acid sequence coverage for the identified proteins; *^j^*: The number of unique peptides identified for each protein; *^k^*: The mean values of protein spot volumes relative to total volume of all the spots. Five samples (from left to right are YLM, YLC, MLM, MLC, and LENF) of various leaf regions at different development stages of *Hosta* “Gold Standard”. YLM, young leaf margin; YLC, young leaf center; MLM, mature leaf margin; MLC, mature leaf center; LENF, leaf under excess nitrogen fertilization. The different small letters on the columns indicate significant differences (*p* < 0.05) among the five samples as determined by one-way Analysis of Variance (ANOVA). Error bars indicate ± standard error (SE).

## Supplementary Materials

Supplementary materials can be found at http://www.mdpi.com/1422-0067/17/3/346/s1.

## References

[1] Kumar A.M., Söll D. (2000). Antisense *HEMA1* RNA expression inhibits heme and chlorophyll biosynthesis in Arabidopsis. Plant Physiol..

[2] Chen M., Choi Y., Voytas D.F., Rodermel S. (2000). Mutations in the Arabidopsis *VAR2* locus cause leaf variegation due to the loss of a chloroplast FtsH protease. Plant J..

[3] Aluru M.R., Bae H., Wu D., Rodermel S.R. (2001). The Arabidopsis immutans mutation affects plastid differentiation and the morphogenesis of white and green sectors in variegated plants. Plant Physiol..

[4] Wu Z., Zhang X., He B., Diao L., Sheng S., Wang J., Guo X., Su N., Wang L., Jiang L. (2007). A chlorophyll-deficient rice mutant with impaired chlorophyllide esterification in chlorophyll biosynthesis. Plant Physiol..

[5] Terry M. (1997). Phytochrome chromophore-deficient mutants. Plant Cell Environ..

[6] Yu F., Fu A., Aluru M., Park S., Xu Y., Liu H., Liu X., Foudree A., Nambogga M., Rodermel S. (2007). Variegation mutants and mechanisms of chloroplast biogenesis. Plant Cell Environ..

[7] Hou D.Y., Xu H., Du G.Y., Lin J.T., Duan M., Guo A.G. (2009). Proteome analysis of chloroplast proteins in stage albinism line of winter wheat (*triticum aestivum*) *FA85*. BMB Rep..

[8] Li Q., Huang J., Liu S., Li J., Yang X., Liu Y., Liu Z. (2011). Proteomic analysis of young leaves at three developmental stages in an albino tea cultivar. Proteome Sci..

[9] Tcherkez G., Guérard F., Gilard F., Lamothe M., Mauve C., Gout E., Bligny R. (2012). Metabolomic characterisation of the functional division of nitrogen metabolism in variegated leaves. Funct. Plant Biol..

[10] Toshoji H., Katsumata T., Takusagawa M., Yusa Y., Sakai A. (2012). Effects of chloroplast dysfunction on mitochondria: White sectors in variegated leaves have higher mitochondrial DNA levels and lower dark respiration rates than green sectors. Protoplasma.

[11] Sakamoto W., Uno Y., Zhang Q., Miura E., Kato Y. (2009). Arrested differentiation of proplastids into chloroplasts in variegated leaves characterized by plastid ultrastructure and nucleoid morphology. Plant Cell Physiol..

[12] Yamasaki H., Heshiki R., Ikehara N. (1995). Leaf-goldenning induced by high light in *Ficus microcarpa* L. f., a tropical fig. J. Plant Res..

[13] Takahashi S., Tamashiro A., Sakihama Y., Yamamoto Y., Kawamitsu Y., Yamasaki H. (2002). High-susceptibility of photosynthesis to photoinhibition in the tropical plant *Ficus microcarpa* L. f. cv. Golden Leaves. BMC Plant Biol..

[14] Deng B. (2012). Antioxidative response of Golden Agave leaves with different degrees of variegation under high light exposure. Acta Physiol. Plant..

[15] Hung C., Xie J. (2009). A comparison of plants regenerated from a variegated *Epipremnum aureum*. Biol. Plant..

[16] Downton W., Grant W. (1994). Photosynthetic and growth responses of variegated ornamental species to elevated CO_2_. Funct. Plant Biol..

[17] Raveh E., Wang N., Nobel P.S. (1998). Gas exchange and metabolite fluctuations in green and yellow bands of variegated leaves of the monocotyledonous CAM species *Agave americana*. Physiol. Plant..

[18] Wang Y., Meng Y.-L., Ishikawa H., Hibino T., Tanaka Y., Nii N., Takabe T. (1999). Photosynthetic adaptation to salt stress in three-color leaves of a C_4_ plant *Amaranthus tricolor*. Plant Cell Physiol..

[19] Liu Y., Zhang J., Li X., Liu H., Sun G. (2011). Effects of nitrogen fertilizer rate on leaf color of chimera *Hosta* “Gold standard”. Acta Prataculturae Sin..

[20] Fulgosi H., Ljubešić N., Wrischer M. (2013). Regreening of yellow leaves. Plastid Development in Leaves during Growth and Senescence.

[21] Yuan M., Xu M.Y., Yuan S., Chen Y.E., Du J.B., Xu F., Zhang Z.W., Guo Z.C., Zhao Z.Y., Lin H.H. (2010). Light regulation to chlorophyll synthesis and plastid development of the chlorophyll-less golden-leaf privet. J. Integr. Plant Biol..

[22] Vaughn K.C., Wilson K.G., Stewart K.D. (1978). Light-harvesting pigment-protein complex deficiency in *Hosta* (Liliaceae). Planta.

[23] Fulgosi H., Jurić S., Lepeduš H., Hazler-Pilepić K., Prebeg T., Ljubešić N. (2008). Thylakoid system disassembly during bleaching of aurea mutants of maple *Acer negundo* Hassk. var. *Odessanum*. Croat. Chem. Acta.

[24] Okabe K., Schmid G.H., Straub J. (1977). Genetic characterization and high efficiency photosynthesis of an aurea mutant of tobacco. Plant Physiol..

[25] Kawata E.E., Cheung A.Y. (1990). Molecular analysis of an aurea photosynthetic mutant (*Su/Su*) in tobacco: LHCP depletion leads to pleiotropic mutant phenotypes. EMBO J..

[26] Oelmüller R., Kendrick R. (1991). Blue light is required for survival of the tomato phytochrome-deficient aurea mutant and the expression of four nuclear genes coding for plastidic proteins. Plant Mol. Biol..

[27] Oelmüller R., Kendrick R., Briggs W. (1989). Blue-light mediated accumulation of nuclear-encoded transcripts coding for proteins of the thylakoid membrane is absent in the phytochrome-deficient aurea mutant of tomato. Plant Mol. Biol..

[28] Palomares R., Herrmann R., Oelmüller R. (1991). Different blue-light requirement for the accumulation of transcripts from nuclear genes for thylakoid proteins in *Nicotiana tabacum* and *Lycopersicon esculentum*. J. Photochem. Photobiol. B Biol..

[29] American Hosta Society (2009). Registrations. Hosta J..

[30] Zhang J., Liu Y., Li X., Liu H., Sun G., He Q. (2011). Effects of excessive application of nitrogen fertilizer on leaf color, key enzymes activities of nitrogen metabolism and chloroplast ultrastructure of a chimera *Hosta* “Gold Standard”. Acta Prataculturae Sin..

[31] Chen S., Harmon A.C. (2006). Advances in plant proteomics. Proteomics.

[32] Yu J., Chen S., Zhao Q., Wang T., Yang C., Diaz C., Sun G., Dai S. (2011). Physiological and proteomic analysis of salinity tolerance in *Puccinellia tenuiflora*. J. Proteome Res..

[33] Suo J., Zhao Q., Zhang Z., Chen S., Cao J., Liu G., Wei X., Wang T., Yang C., Dai S. (2015). Cytological and proteomic analyses of *Osmunda cinnamomea* germinating spores reveal characteristics of fern spore germination and rhizoid tip-growth. Mol. Cell. Proteom..

[34] Zhao Q., Zhang H., Wang T., Chen S., Dai S. (2013). Proteomics-based investigation of salt-responsive mechanisms in plant roots. J. Proteom..

[35] Zhang H., Han B., Wang T., Chen S., Li H., Zhang Y., Dai S. (2011). Mechanisms of plant salt response: Insights from proteomics. J. Proteome Res..

[36] Bernard S.M., Møller A.L.B., Dionisio G., Kichey T., Jahn T.P., Dubois F., Baudo M., Lopes M.S., Tercé-Laforgue T., Foyer C.H. (2008). Gene expression, cellular localisation and function of glutamine synthetase isozymes in wheat (*Triticum aestivum* L.). Plant Mol. Biol..

[37] Joshi R., Karan R., Singla-Pareek S.L., Pareek A. (2016). Ectopic expression of Pokkali phosphoglycerate kinase-2 (OsPGK2-P) improves yield in tobacco plants under salinity stress. Plant Cell Rep..

[38] Lu W., Tang X., Huo Y., Xu R., Qi S., Huang J., Zheng C., Wu C.-A. (2012). Identification and characterization of fructose 1,6-bisphosphate aldolase genes in *Arabidopsis* reveal a gene family with diverse responses to abiotic stresses. Gene.

[39] Sung D.Y., Vierling E., Guy C.L. (2001). Comprehensive expression profile analysis of the Arabidopsis Hsp70 gene family. Plant Physiol..

[40] Weisberg L.A., Wimmers L.E., Turgeon R. (1988). Photoassimilate-transport characteristics of nonchlorophyllous and green tissue in variegated leaves of *Coleus blumei* Benth. Planta.

[41] Joshi P., Nayak L., Misra A.N., Biswal B. (2013). Response of mature, developing and senescing chloroplasts to environmental stress. Plastid Development in Leaves during Growth and Senescence.

[42] Tischner R. (2000). Nitrate uptake and reduction in higher and lower plants. Plant Cell Environ..

[43] Bernard S.M., Habash D.Z. (2009). The importance of cytosolic glutamine synthetase in nitrogen assimilation and recycling. New Phytol..

[44] McNally S.F., Hirel B., Gadal P., Mann A.F., Stewart G.R. (1983). Glutamine synthetases of higher plants evidence for a specific isoform content related to their possible physiological role and their compartmentation within the leaf. Plant Physiol..

[45] Thomsen H.C., Eriksson D., Møller I.S., Schjoerring J.K. (2014). Cytosolic glutamine synthetase: A target for improvement of crop nitrogen use efficiency?. Trends Plant Sci..

[46] Pérez-García A., de Vicente A., Cantón F.R., Cazorla F.M., Codina J.C., García-Gutiérrez Á., Cánovas F.M. (1998). Light-dependent changes of tomato glutamine synthetase in response to *Pseudomonas syringae* infection or phosphinothricin treatment. Physiol. Plant..

[47] Finnemann J., Schjoerring J.K. (2000). Post-translational regulation of cytosolic glutamine synthetase by reversible phosphorylation and 14-3-3 protein interaction. Plant J..

[48] Santos C., Pereira A., Pereira S., Teixeira J. (2004). Regulation of glutamine synthetase expression in sunflower cells exposed to salt and osmotic stress. Sci. Hortic. Amst..

[49] Tavernier V., Cadiou S., Pageau K., Laugé R., Reisdorf-Cren M., Langin T., Masclaux-Daubresse C. (2007). The plant nitrogen mobilization promoted by *Colletotrichum lindemuthianum* in *Phaseolus* leaves depends on fungus pathogenicity. J. Exp. Bot..

[50] Borner T., Mendel R., Schiemann J. (1986). Nitrate reductase is not accumulated in chloroplast-ribosome-deficient mutants of higher plants. Planta.

[51] Kozaki A., Sakamoto A., Takeba G. (1992). The promoter of the gene for plastidic glutamine synthetase (GS2) from rice is developmentally regulated and exhibits substrate-induced expression in transgenic tobacco plants. Plant Cell Physiol..

[52] Cren M., Hirel B. (1999). Glutamine synthetase in higher plants regulation of gene and protein expression from the organ to the cell. Plant Cell Physiol..

[53] Sukanya R., Li M.-G., Snustad D.P. (1994). Root-and shoot-specific responses of individual glutamine synthetase genes of maize to nitrate and ammonium. Plant Mol. Biol..

[54] Ishiyama K., Inoue E., Watanabe-Takahashi A., Obara M., Yamaya T., Takahashi H. (2004). Kinetic properties and ammonium-dependent regulation of cytosolic isoenzymes of glutamine synthetase in Arabidopsis. J. Biol. Chem..

[55] Ishiyama K., Inoue E., Tabuchi M., Yamaya T., Takahashi H. (2004). Biochemical background and compartmentalized functions of cytosolic glutamine synthetase for active ammonium assimilation in rice roots. Plant Cell Physiol..

[56] Bondada B.R., Syvertsen J.P. (2003). Leaf chlorophyll, net gas exchange and chloroplast ultrastructure in citrus leaves of different nitrogen status. Tree Physiol..

[57] Yang H., Xia X., Fang W., Fu Y., An M., Zhou M. (2015). Identification of genes involved in spontaneous leaf color variation in *Pseudosasa japonica*. Genet. Mol. Res..

[58] Zavaleta-Mancera H., Franklin K., Ougham H., Thomas H., Scott I. (1999). Regreening of senescent *Nicotiana* leaves I. Reappearance of NADPH-protochlorophyllide oxidoreductase and light-harvesting chlorophyll *a*/*b*-binding protein. J. Exp. Bot..

[59] Plücken H., Müller B., Grohmann D., Westhoff P., Eichacker L.A. (2002). The HCF136 protein is essential for assembly of the photosystem II reaction center in *Arabidopsis thaliana*. FEBS Lett..

[60] Yamamoto Y. (2001). Quality control of photosystem II. Plant Cell Physiol..

[61] Hemmingsen S.M. (1990). The plastid chaperonin. Semin. Cell Biol..

[62] Gutteridge S., Gatenby A.A. (1995). Rubisco synthesis, assembly, mechanism, and regulation. Plant Cell.

[63] Suzuki K., Nakanishi H., Bower J., Yoder D.W., Osteryoung K.W., Miyagishima S.-Y. (2009). Plastid chaperonin proteins Cpn60α and Cpn60β are required for plastid division in *Arabidopsis thaliana*. BMC Plant Biol..

[64] Raines C.A., Lloyd J.C., Dyer T.A. (1991). Molecular biology of the C_3_ photosynthetic carbon reduction cycle. Photosynth. Res..

[65] Raines C.A. (2003). The Calvin cycle revisited. Photosynth. Res..

[66] Zhang J., Zhou M., Li X., Yu X., Jiang C., Sun G. (2007). The single and interactive effects of nitrogen application rate and light condition on *Hosta* “Blue Umbrella” growth and photosynthetic characteristics. Acta Hortic. Sin..

[67] Zhou M., Zhang J., Li M., Wang Z. (2007). The single and interactive effects of nitrogen application rate and light condition on *Hosta* “Francee” growth and photosynthetic characteristics. J. Hebei For. Sci. Technol..

[68] Hikosaka K. (2004). Interspecific difference in the photosynthesis-nitrogen relationship: patterns, physiological causes, and ecological importance. J. Plant Res..

[69] Luo J., Zhou J., Li H., Shi W., Polle A., Lu M., Sun X., Luo Z.-B. (2015). Global poplar root and leaf transcriptomes reveal links between growth and stress responses under nitrogen starvation and excess. Tree Physiol..

[70] Mokhele B., Zhan X., Yang G., Zhang X. (2012). Review: Nitrogen assimilation in crop plants and its affecting factors. Can. J. Plant Sci..

[71] Guo S., Zhou Y., Gao Y., Li Y., Shen Q. (2007). New insights into the nitrogen form effect on photosynthesis and photorespiration. Pedosphere.

[72] Pogson B.J., Albrecht V. (2011). Genetic dissection of chloroplast biogenesis and development: An overview. Plant Physiol..

[73] Albrecht V., Ingenfeld A., Apel K. (2006). Characterization of the *snowy cotyledon 1* mutant of *Arabidopsis thaliana*: the impact of chloroplast elongation factor G on chloroplast development and plant vitality. Plant Mol. Biol..

[74] Wu W., Zhu Y., Ma Z., Sun Y., Quan Q., Li P., Hu P., Shi T., Lo C., Chu I.K. (2013). Proteomic evidence for genetic epistasis: ClpR4 mutations switch leaf variegation to virescence in Arabidopsis. Plant J..

[75] Kato Y., Sakamoto W. (2013). Plastid protein degradation during leaf development and senescence: Role of proteases and chaperones. Plastid Development in Leaves during Growth and Senescence.

[76] Su P.-H., Li H.-M. (2010). Stromal Hsp70 is important for protein translocation into pea and Arabidopsis chloroplasts. Plant Cell.

[77] Latijnhouwers M., Xu X.-M., Møller S.G. (2010). Arabidopsis stromal 70-kDa heat shock proteins are essential for chloroplast development. Planta.

[78] Lee U., Rioflorido I., Hong S.W., Larkindale J., Waters E.R., Vierling E. (2007). The Arabidopsis ClpB/Hsp100 family of proteins: chaperones for stress and chloroplast development. Plant J..

[79] Sjögren L.L., MacDonald T.M., Sutinen S., Clarke A.K. (2004). Inactivation of the *clpC1* gene encoding a chloroplast Hsp100 molecular chaperone causes growth retardation, leaf chlorosis, lower photosynthetic activity, and a specific reduction in photosystem content. Plant Physiol..

[80] Zaltsman A., Ori N., Adam Z. (2005). Two types of FtsH protease subunits are required for chloroplast biogenesis and photosystem II repair in Arabidopsis. Plant Cell.

[81] Cederholm S.N., Bäckman H.G., Pesaresi P., Leister D., Glaser E. (2009). Deletion of an organellar peptidasome PreP affects early development in *Arabidopsis thaliana*. Plant Mol. Biol..

[82] Goloubinoff P., Mogk A., Zvi A.P.B., Tomoyasu T., Bukau B. (1999). Sequential mechanism of solubilization and refolding of stable protein aggregates by a bichaperone network. PNAS.

[83] Clarke A.K., MacDonald T.M., Sjögren L.L. (2005). The ATP-dependent Clp protease in chloroplasts of higher plants. Physiol. Plant..

[84] Guo F., Maurizi M.R., Esser L., Xia D. (2002). Crystal structure of ClpA, an Hsp100 chaperone and regulator of ClpAP protease. J. Biol. Chem..

[85] Nakagawara E., Sakuraba Y., Yamasato A., Tanaka R., Tanaka A. (2007). Clp protease controls chlorophyll *b* synthesis by regulating the level of chlorophyllide *a* oxygenase. Plant J..

[86] Yamasato A., Nagata N., Tanaka R., Tanaka A. (2005). The N-terminal domain of chlorophyllide *a* oxygenase confers protein instability in response to chlorophyll *b* accumulation in Arabidopsis. Plant Cell.

[87] Kmiec B., Glaser E. (2012). A novel mitochondrial and chloroplast peptidasome, PreP. Physiol. Plant..

[88] Hao L.S.M.F. (1999). Studies on the stage albescent phenomenon in tea the changes of rubpcase and proteinase. Sci. Agric. Sin..

[89] Buchanan-Wollaston V., Earl S., Harrison E., Mathas E., Navabpour S., Page T., Pink D. (2003). The molecular analysis of leaf senescence—A genomics approach. Plant Biotechnol. J..

[90] Olinares P.D.B., Kim J., van Wijk K.J. (2011). The Clp protease system: A central component of the chloroplast protease network. BBA Bioenerg..

[91] Liu X., Yu F., Rodermel S. (2010). Arabidopsis chloroplast FtsH, *var2* and suppressors of *var2* leaf variegation: A review. J. Integr. Plant Biol..

[92] Mayer M., Bukau B. (2005). Hsp70 chaperones: cellular functions and molecular mechanism. Cell. Mol. Life Sci..

[93] Ling Q., Trösch R., Jarvis P. (2013). The ins and outs of chloroplast protein transport. Plastid Development in Leaves during Growth and Senescence.

[94] Rial D.V., Arakaki A.K., Ceccarelli E.A. (2000). Interaction of the targeting sequence of chloroplast precursors with Hsp70 molecular chaperones. Eur. J. Biochem..

[95] Waegemann K., Paulsen H., Soll J. (1990). Translocation of proteins into isolated chloroplasts requires cytosolic factors to obtain import competence. FEBS Lett..

[96] Kourtz L., Ko K. (1997). The early stage of chloroplast protein import involves Com70. J. Biol. Chem..

[97] Kessler F., Blobel G. (1996). Interaction of the protein import and folding machineries of the chloroplast. PNAS.

[98] Zhang J., Shi L., Shi A., Zhang Q. (2004). Photosynthetic responses of four *Hosta* cultivars to shade treatments. Photosynthetica.

[99] Willis O.O., Mouti M.E., Sila D.N., Mwasaru M., Thiongo G., Murage H., Ojijo N.O. (2013). Physico-chemical properties and antioxidant potential of syrup prepared from “Madhura”sweet sorghum (*Sorghum bicolor* L. Moench) cultivar grown at different locations in Kenya. Sugar Tech..

[100] Arnon D.I. (1949). Copper enzymes in isolated chloroplasts. Polyphenoloxidase in *Beta vulgaris*. Plant Physiol..

[101] Lichtenthaler H., Wellburn A. (1983). Determinations of total carotenoids and chlorophylls a and b of leaf extracts in different solvent. Biochem. Soc. Trans..

[102] Zhao S., Shi G., Dong X. (2002). Experiment Instruction of Plant Physiology.

[103] Singh R.P., Srivastava H.S. (1986). Increase in glutamate synthase (NADH) activity in maize seedlings in response to nitrate and ammonium nitrogen. Physiol. Plant..

[104] Wang X., Chen S., Zhang H., Shi L., Cao F., Guo L., Xie Y., Wang T., Yan X., Dai S. (2010). Desiccation tolerance mechanism in resurrection fern-ally *Selaginella tamariscina* revealed by physiological and proteomic analysis. J. Proteome Res..

[105] Dai S., Li L., Chen T., Chong K., Xue Y., Wang T. (2006). Proteomic analyses of *Oryza sativa* mature pollen reveal novel proteins associated with pollen germination and tube growth. Proteomics.

[106] Sun Y., Wang Q., Li Z., Hou L., Dai S., Liu W. (2013). Comparative proteomics of peanut gynophore development under dark and mechanical stimulation. J. Proteome Res..

[107] National Center for Biotechnology Information non-redundant (NCBInr) protein databases. http://www.ncbi.nlm.nih.gov/protein/.

[108] Matrix Science. http://www.matrixscience.com.

[109] UniProt. http://www.uniprot.org/.

[110] Zhao Q., Gao J., Suo J., Chen S., Wang T., Dai S. (2015). Cytological and proteomic analyses of horsetail (*Equisetum arvense* L.) spore germination. Front. Plant Sci..

[111] YLoc: Interpretable Subcellular Localization Prediction. http://abi.inf.uni-tuebingen.de/Services/YLoc/webloc.cgi.

[112] LocTree3: Protein Subcellular Localization Prediction System. https://rostlab.org/services/loctree3/.

[113] Plant-mPLoc: Predicting Subcellular Localization of Plant Proteins Including Those with Multiple Sites. http://www.csbio.sjtu.edu.cn/bioinf/plant-multi/#.

[114] ngLOC: A Bayesian Method for Predicting Protein Subcellular Localization. http://genome.unmc.edu/ngLOC/index.html.

[115] TargetP 1.1 Server. http://www.cbs.dtu.dk/services/TargetP/.

[116] BoxShade: Pretty Printing and Shading of Multiple-Alignment Files. http://www.ch.embnet.org/software/BOX_form.html.

[117] Cluster 3.0 Software. http://bonsai.hgc.jp/~mdehoon/software/cluster/software.htm.

[118] Yu J., Chen S., Wang T., Sun G., Dai S. (2013). Comparative proteomic analysis of *Puccinellia tenuiflora* leaves under Na_2_CO_3_ stress. Int. J. Mol. Sci..

