# Supplementary material for: Proteomic Analysis Reveals the Leaf Color Regulation Mechanism in Chimera Hosta “Gold Standard” Leaves
Source: Int J Mol Sci. 2016 Mar 8;17(3):346. doi: 10.3390/ijms17030346 (PMC4813207; doi:10.3390/ijms17030346)
Supplement: Supplementary file 1 [file ijms-17-00346-s001.zip › ijms-114209-Supplementary Materials/ijms-114209-supplementary.pdf]

Supplementary Materials: The Leaf Color Regulation Mechanism in Chimera *Hosta* "Gold Standard" Leaves Revealed from Proteomic Analysis

Juanjuan Yu, Jinzheng Zhang, Qi Zhao, Yuelu Liu, Sixue Chen, Hongliang Guo, Lei Shi and Shaojun Dai

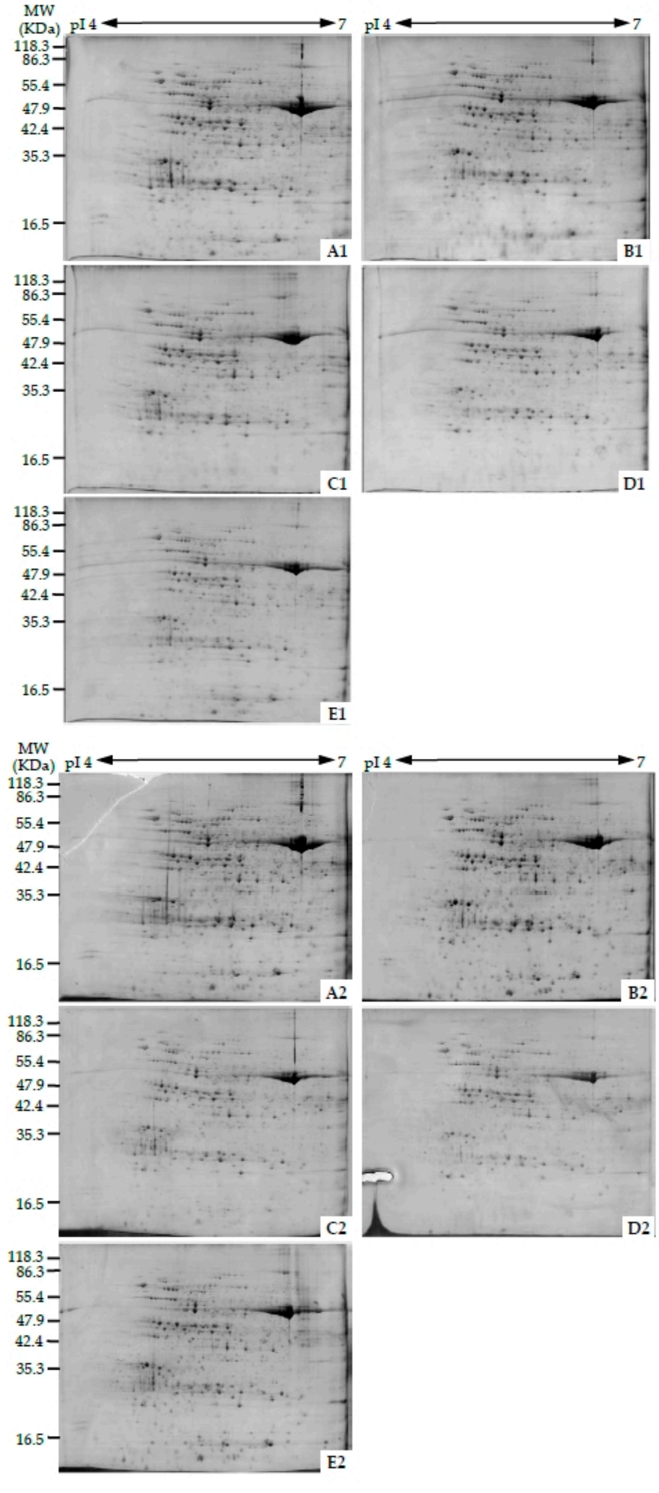

Figure S1. *Cont.*

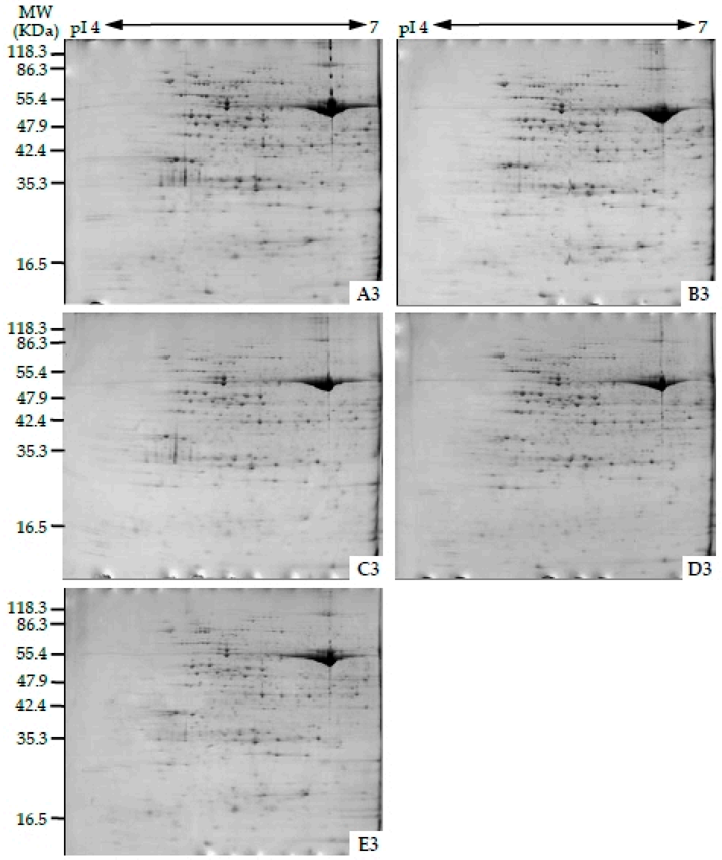

Figure S1. Three biological replicates of 2-DE gels of proteins extracted from various leaf regions in different development stages of *Hosta* "Gold Standard". (**A1**–**A3**) young leaf margin (YLM); (**B1**–**B3**) young leaf center (YLC); (**C1**–**C3**) mature leaf margin (MLM); (**D1**–**D3**) mature leaf center (MLC); (**E1**–**E3**) leaf under excess nitrogen fertilization (LENF). Proteins were separated on 24 cm IPG strips (pH 4–7 linear gradient) using isoelectric focusing (IEF) in the first dimension, followed by 12.5% SES-PAGE gels in the second dimension. The 2-DE gel was stained with Coomassie Brilliant Blue. Molecular weight (*M*w) in kilodaltons (kDa) and pI of proteins are indicated on the left and top of the gel, respectively.

```
gi|71361904|spot 795|GS1c  MALLTDLLNLDLTDSTEKIIAEYIWIGGSGMDLRSKARTLPGPVTDPSKLPKWNYDGSSTGQAPGEDSEVILYPQAIFKD   80
gi|71361904|spot 806|GS1c  MALLTDLLNLDLTDSTEKIIAEYIWIGGSGMDLRSKARTLPGPVTDPSKLPKWNYDGSSTGQAPGEDSEVILYPQAIFKD   80
consensus                  malltdllnldltdstekiiaeyiwiggsgmdlrskartlpgpvtdpsklpkwnydgsstgqapgedsevilypqaifkd

gi|71361904|spot 795|GS1c  PFRKGNNILVMCDCYTPAGVPIPTNKRYNAVKIFSNPDVAKEEPWYGIEQEYTLLQKDINWPLGWPVGGFPGPQGPYYCS  160
gi|71361904|spot 806|GS1c  PFRKGNNILVMCDCYTPAGVPIPTNKRYNAVKIFSNPDVAKEEPWYGIEQEYTLLQKDINWPLGWPVGGFPGPQGPYYCS  160
consensus                  pfrkgnnilvmcdcytpagvpiptnkrynavkifsnpdvakeepwygieqeytllqkdinwplgwpvggfpgpqgpyycs

gi|71361904|spot 795|GS1c  IGADKSFGRDIVDSHYKACLFAGVNISGINGEVMPGQWEFQVGPTVGISAGDQVWVARYLLERITEIAGVVVTFDPKPIP  240
gi|71361904|spot 806|GS1c  IGADKSFGRDIVDSHYKACLFAGVNISGINGEVMPGQWEFQVGPTVGISAGDQVWVARYLLERITEIAGVVVTFDPKPIP  240
consensus                  igadksfgrdivdshykaclfagvnisgingevmpgqwefqvgptvgisagdqvwvaryllerionalgvvvtfdpkpip

gi|71361904|spot 795|GS1c  GDWNGAGAHTNYSTESMRKDGGFKVIVDAVEKLKLKHKEHIAAYGEGNERRLTGKHETADINTFSWGVANRGASVRVGRE  320
gi|71361904|spot 806|GS1c  GDWNGAGAHTNYSTESMRKDGGFKVIVDAVEKLKLKHKEHIAAYGEGNERRLTGKHETADINTFSWGVANRGASVRVGRE  320
consensus                  gdwngagahtnystesmrkdggfkvivdaveklklkhkehiaaygegnerrltgkhetadintfswgvanrgasvrvgre

gi|71361904|spot 795|GS1c  TEQNGKGYFEDRRPASNMDPYVVTSMIAETTILWK                                              355
gi|71361904|spot 806|GS1c  TEQNGKGYFEDRRPASNMDPYVVTSMIAETTILWK                                              355
consensus                  teqngkgyfedrrpasnmdpyvvtsmiaettilwk
```

A

```
gi|194396261|spot 338|TK  MASSSSLTLSQAILSRSVPRHGSASSSQLSPSSLTFSGLKSNPNITTSRRRTPSSAAAAAVVRSPAIRASAATETIEKTE   80
gi|224063766|spot 346|TK  MASTSSLTLSQALLARAVSHNAIDNLRDSRLSLVSLPAFSGLKSTTCTATRATTTTSRRRRVSSRQVRAAAVETLDATTE   80
consensus                 mas ssltlsqa l r v              s                t    r           v s   ra a        te

gi|194396261|spot 338|TK  TALVDKSVNTIRFLAIDAVEKANSGHPGLPMGCAPMGHILYDEVMRYNPKNPYWFNRDRFVLSAGHGCMLQYALLHLAGY  160
gi|224063766|spot 346|TK  TSLVEKSVNTIRFLAIDAVEKANSGHPGLPMGCAPMGHILYDEVMRYNPKNPYWFNRDRFVLSAGHGCMLQYALLHLAGY  160
consensus                 t lv ksvntirflaidavekansghpglpmgcapmghilydevmrynpknpywfnrdrfvlsaghgcmlqyallhlagy

gi|194396261|spot 338|TK  DAVREEDLKSFRQWGSKTPGHPENFETPGVEVTTGPLGQGIANAVGLALVEKHLAARFNKPDAEIVDHYTYVILGDGCQM  240
gi|224063766|spot 346|TK  DSVKEEDLKSFRQWGSRTPGHPENFETPGVEVTTGPLGQGIANAVGLALAEKHLAARYNKPDNEIVDHYTYVILGDGCQM  240
consensus                 d v eedlksfrqwgs tpghpenfetpgvevttgplgqgianavglal ekhlaar nkpd eivdhytyvilgdgcqm

gi|194396261|spot 338|TK  EGISQEACSLAGHWGLGKLIAFYDDNHISIDGDTEIAFTEDVGARFEALGWHVIWVKNGNTGYDEIRAAIKEAKTVTDKP  320
gi|224063766|spot 346|TK  EGISNEACSLAAHWGLGKLIAFYDDNHISIDGDTEIAFTEDVDKRFEGLGWHVIWVKNGNTGYDEIRAAIKEAKSVKDKP  320
consensus                 egis eacsla hwglgkliafyddnhisidgdteiaftedv  rfe lgwhviwvkngntgydeiraaikeak v dkp

gi|194396261|spot 338|TK  TMIKVTTTIGFGSPNKANSYSVHGSALGAKEVEATRSNLGWPYEPFHVPEDVKSHWSRHVTEGAALEAGWNTKFAEYEKK  400
gi|224063766|spot 346|TK  TLIKVTTTIGYGSPNKANSYSVHGSALGAKEVDATRQNLGWPYEPFHVPEDVKQHWSRHTPAGAAFEAEWNAKFAEYEKK  400
consensus                 t ikvtttig gspnkansysvhgsalgakev atr nlgwpyepfhvpedvk hwsrh   gaa ea wn kfaeyekk

gi|194396261|spot 338|TK  YPEEAAELKSITTGELPAGWEKALPTYTPESPADATRNLSQQNLNALVKVLPGFLGGSADLASSNMTLMKMFGDFQKNTP  480
gi|224063766|spot 346|TK  YSEEAAELKSIANGELPAGWEKALPTYTPESPADATRNLSQQNLNALVKVLPGLLGGSADLASSNMTLLKMFGDFQKDTP  480
consensus                 y eeaaelksi  gelpagwekalptytpespadatrnlsqqnlnalvkvlpg lggsadlassnmtl kmfgdfqk tp

gi|194396261|spot 338|TK  EERNLRFGVREHGMGAICNGIALHSPGLIPYCATFFVFTDYMRGAMRISALSEAGVIYVMTHDSIGLGEDGPTHQPIEHL  560
gi|224063766|spot 346|TK  EERNVRFGVREHGMGAICNGIALHCPGLIPYCATFFVFTDYMRAAMRISALSEAGVIYVMTHDSIGLGEDGPTHQPIEHL  560
consensus                 eern rfgvrehgmgaicngialh pglipycatffvftdymr amrisalseagviyvmthdsiglgedgpthqpiehl

gi|194396261|spot 338|TK  ASFRAMPNILMFRPADGNETAGAYKVAVLKWKTPSILALSRQKLPQLAGSSIEGAAKGGYILSDNSSGNKPDVILIGTGS  640
gi|224063766|spot 346|TK  ASFRAMPNILMLRPADGNETAGAYKVAVLNRKRPSILALSRQKLPQLPGTSIEGVEKGGYIISDNSSSNKPDVILIGTGS  640
consensus                 asframpnilm rpadgnetagaykvavl  k psilalsrqklpql g sieg  kggyi sdnss nkpdviligtgs

gi|194396261|spot 338|TK  ELEIAVKAADELRKEGKAVRVVSFVCWELFEEQSADYKESVLPSSVTARVSIEAGSTFGWEKYVGSKGKAIGIDRWGASA  720
gi|224063766|spot 346|TK  ELEIAAKAAEELRKEGKAVRVVSFVSWELFDEQSDAYKESVLPAAVEARVSIEAGSTFGWEKIVGDKGKAIGIDRFGASA  720
consensus                 eleia kaa elrkegkavrvvsfv welf eqs  ykesvlp  v arvsieagstfgwek vg kgkaigidr gasa

gi|194396261|spot 338|TK  PAGKIYKEYGITAEAVVAAAKQV                                                          743
gi|224063766|spot 346|TK  PAGKIYKEYGITTEAVIAAAKEV                                                          743
consensus                 pagkiykeygit eav aaak v
```

B

Figure S2. *Cont.*

```
gi|242090109|spot 459|RBP-α  ..........................MASANAISTASLISPLSQ.GRARRARNGRSQRFVVRAEAKDIAFDQK..SRAAL   51
gi|223948025|spot 472|RBP-β  ............................MASTFGATSTVGLMAA.PTGKNVRLQRRANFRVK.AAKELYFNKDGSAIKKL   50
gi|225442531|spot 476|RBP-β  MASSFATMSSIGYFTSSSSHTMDKKFLGSSRRLSSFASISANSFEGRKQSMVLQKRCSPKVRAMAKELYFNKDGSAIKKL   80
consensus                                                                                v   ak   f        l

gi|242090109|spot 459|RBP-α  QAGVEKLANAVGVTLGPRGRNVVLD.EYGSPKVVNDGVTIARAIELYDPMENAGAALIREVASKTNDSAGDGTTTASVLA  130
gi|223948025|spot 472|RBP-β  QTGVNKLADLVGVTLGPKGRNVVLESKYGSPKIVNDGVTVAREVELEDPVENIGAKLVRQAAAKTNDLAGDGTTTSVVLA  130
gi|225442531|spot 476|RBP-β  QTGVNKLADLVGVTLGPKGRNVVLESKYGSPKIVNDGVTVAREVELEDPVENIGARLVRQAASKTNDLAGDGTTTSVVLA  160
consensus                    q gv kla  vgvtlgp grnvvl   ygspk vndgvt ar  el dp en ga l r  a ktnd agdgttt  vla

gi|242090109|spot 459|RBP-α  REIIKLGLLSVTSGANPVSLKKGIDKTVQGLIQELENKARPVKGGGDIKAVASISAGNDEFIGSMIAEAIDKVGPDGVLS  210
gi|223948025|spot 472|RBP-β  QGLIAEGVKVVAAGANPVQITRGIEKTAKALVEELRKLSKEVEDS.ELADVAAVSAGNNYEIGNMIAEAMSKVGRKGVVT  209
gi|225442531|spot 476|RBP-β  QGLITEGVKVVAAGANPVQITRGIENTTKALVAELKLMSKEVEDS.ELADVAAVSAGNNYEVGYMIAEAMGQVGRKGVVT  239
consensus                       i g  v ganpv    gi t   l el       v         va  sagn    g miaea   vg  gv

gi|242090109|spot 459|RBP-α  IESSSSFETTVEVEEGMEIDRGYISPQFVTNLEKSIVEFENAKVLITDQKISSIKEILPILEKTTQLRAPLFIIAEDITG  290
gi|223948025|spot 472|RBP-β  LEEGKSSENFLYVVEGMQFDRGYISPYFVTDSEKMTAEYENCKLLLVDKKITNARDLINVLEEAIRGAYPILIIAEDIEQ  289
gi|225442531|spot 476|RBP-β  LEEGKSAENNLYVVEGMQFDRGYISPYFVTDSEKMAVEYENCKLLLVDKKITNARDLINVLEDAIKGGYPILIIAEDIEQ  319
consensus                     e   s e    v egm  drgyisp fvt  ek    e en k l  d ki          le        p  iiaedi

gi|242090109|spot 459|RBP-α  EALATLVVNKLRGILNVAAIKAPSFGERRKAVLQDIAIVTGAEFLAKDLGLLVENATEAQLGTARKVTIHQTTTLIADA  370
gi|223948025|spot 472|RBP-β  EALATLVVNKLRGSLKIAAIKAPGFGERKTQYLDDIAILTGATVIRDEVGLSLDKADKSVLGTAAKVVLTKESTTIVGDG  369
gi|225442531|spot 476|RBP-β  EALATLVVNKLRGALKIAALKAPGFGERKSQYLDDIAILTGGTVIREEVGLSLDKAEKEVLGHAAKVVLTKDTTTIVGDG  399
consensus                    ealatlvvnklrg l  aa kap fger    l diai tg        gl    a    lg a kv       tt   d

gi|242090109|spot 459|RBP-α  ASKDEIQARVAQLKKELAETDSVYDTEKLAERIAKLAGGVAVIKVGAATETELEDRQLRIEDAKNATFAAIEEGIVPGGG  450
gi|223948025|spot 472|RBP-β  STQEEVTKRVAQIKNLIEAAEQEYEKEKLNERIAKLAGGVAVIQVGAQTETELKEKKLRVEDALNATKAAVEEGIVVGGG  449
gi|225442531|spot 476|RBP-β  STQEAVNKRVAQIRNLVEAADQDYEKEKLNERIAKLSGGVAVIQVGAQTETELKEKKLRVEDALNATKAAVEEGIVVGGG  479
consensus                            rvaq           y  ekl eriakl ggvavi vga tetel     lr eda nat aa eegiv ggg

gi|242090109|spot 459|RBP-α  TAYVHLSTVVPSIKEKIEDPDERLGADIIQKALVAPASLIAHNAGVEGEVVVEKIKDS.VWEVGYNAMTDTYENLIEAGV  529
gi|223948025|spot 472|RBP-β  CTLLRLAAKVDAIKDTLENDEQKVGAEIVRRALSYPLKLIAKNAGVNGSVVTEKVLSNDNFKYGYNAATGQYEDLMAAGI  529
gi|225442531|spot 476|RBP-β  CTLLRLAAKVDAIKDTLDSDEQKVGADIVKRALSYPMKLIAKNAGVNGSVVIEKVLSSDNPKYGYNAATGKYEDLMAAGI  559
consensus                         l   v  ik           ga i   al  p  lia nagv g vv ek         gyna t  ye l  ag

gi|242090109|spot 459|RBP-α  IDPAKVTRCALQNAASVAGMVLTTQAIVVEKPKPKPQ.VAEPSEGSLAV                                577
gi|223948025|spot 472|RBP-β  IDPTKVVRCCLEHAASVAKTFLTSDVVVVDIKEPEAAPVANPMDNSGYG                                578
gi|225442531|spot 476|RBP-β  IDPTKVVRCCLEHAASVARTFLTSDAVVVDIKEPEPIPAGNPMDNSGYG                                608
consensus                    idp kv rc l  aasva   lt    vv    p       p   s
```

C

```
gi|255646270|spot 832|PRK  MAACTVYSTQSLSTNCSISTPSKTQLSFFHQKQVVFWRNSKKGSCSSRRSYVITCAAGDSQTVVIGLAADSGCGKSTFMR   80
gi|1885326  |spot 840|PRK  ........................................................AGDSQTIVIGLAADSGCGKSTFMR   24
consensus                                                                          agdsqt viglaadsgcgkstfmr

gi|255646270|spot 832|PRK  RLTSVFGGAAEPPKGGNPDSNTLISDTTTVICLDDYHSLDRTGRKEKGVTALDPRANDFDLMYEQVKALKDGIAVEKPIY  160
gi|1885326  |spot 840|PRK  RLTSVFGGAAEPPKGGNPDSNTLISDTTTVICLDDYHSLDRTGRKEKGVTALDPKANDFDLMYEQVKAIKDGKSVQKPIY  104
consensus                  rltsvfggaaeppkggnpdsntlisdtttvicl ddyhsldrtgrkekgvtaldp andfdlmyeqvka kdg  v kpiy

gi|255646270|spot 832|PRK  NHVTGLLDPPELIKPPKILVIEGLHPMFDSRVRDLLDFSIYLDISNEVKFAWKIQRDMAERGHSLESIKASIEARKPDFE  240
gi|1885326  |spot 840|PRK  NHVTGLLDAPELIKPPKILVIEGLHPMYDSRVRELLDFSIYLDISNEVKFAWKIQRDMAERGHSLESIKASIEARKPDFE  184
consensus                  nhvtglld pelikppkilvieglhpm dsrvr lldfsiyldisnevkfawkiqrdmaerghslesikasiearkpdfe

gi|255646270|spot 832|PRK  AYIDPQKQYADAVIEVLPTQLIPDDNEGKILRVRLIQKEGVKYFSPVYLFDDGSTISWIPCGRKLTCSYPGIKFFYGPET  320
gi|1885326  |spot 840|PRK  AYIDPQKQYADAVIEVLPTQLIPDDNEGKILRVRLIQKAGVKYFSPVYLFDEGSTISWIPCGRKLTCSYPGIKFFYGPET  264
consensus                  ayidpqkqyadavievlptqlipddnegkilrvrliqk gvkyfspvylfd gstiswipcgrkltcsypgikffygpet

gi|255646270|spot 832|PRK  YYGNEVLVVEMDGQFDRLDELIYVESHLSNLSTKFYGEVTQQMLKHADFPGSNNGTGFFQTIVGLKIRDLYEQLIASKAE  400
gi|1885326  |spot 840|PRK  YKGNEVSVVEMDGQFDRLDELIYVESHLSNLSSKFYGEVTQQMLKHADFPGSNNGTGLFQTIVGLKIRDLFEQIVASRAE  344
consensus                  y gnev vvemdgqfdrldeliyveshlsnls kfygevtqqmlkhadfpgsnngtg fqtivglkirdl eq  as ae

gi|255646270|spot 832|PRK  TPVAAKA                                                                           407
gi|1885326  |spot 840|PRK  TPVGAAK                                                                           351
consensus                  tpv a
```

D

Figure S2. *Cont.*

```
gi|225449497|spot 355|HSC70-1  MAGKGE......GPAIGIDLGTTYSCVGVWQHDRVEIIANDQGNRTTPSYVAFTDT.ERLIGDAAKNQVAMNPINTVFDA   73
gi|225449497|spot 361|HSC70-1  MAGKGE......GPAIGIDLGTTYSCVGVWQHDRVEIIANDQGNRTTPSYVAFTDT.ERLIGDAAKNQVAMNPINTVFDA   73
gi|255573627|spot 358|HSC70-2  MAGKGE......GPAIGIDLGTTYSCVGVWQHDRVEIIANDQGNRTTPSYVAFTDT.ERLIGDAAKNQVAMNPVNTVFDA   73
gi|225434984|spot 367|HSC70-2  MAGKGE......GPAIGIDLGTTYSCVGVWQHDRVEIIANDQGNRTTPSYVAFTDT.ERLIGDAAKNQVAMNPINTVFDA   73
gi|147805297|spot 332|cpHSP70  MVRKKASFHVVNEKVVGIDLGTTNSAVAAMEGGKPTIVTNAEGQRTTPSVVAYTKNGDMLVGQIAKRQAVVNPENTFFSV   80
consensus                      m  k            gidlgtt s v         i  n  g rttps va t     l g  ak q   np nt f

gi|225449497|spot 355|HSC70-1  KRLIGRRFSDASVQSDIKHWSFKVVPGPGDKPMITVTYKGEDKQFAAEEISSMVLIKMREIAEAYLGSTVKNAVVTVPAY  153
gi|225449497|spot 361|HSC70-1  KRLIGRRFSDASVQSDIKHWSFKVVPGPGDKPMITVTYKGEDKQFAAEEISSMVLIKMREIAEAYLGSTVKNAVVTVPAY  153
gi|255573627|spot 358|HSC70-2  KRLIGRRFTDASVQSDIKLWPFKVIPGPGDKPMIIVSYKGEDKQFAAEEISSMVLIKMREIAEAYLGSTVKNAVVTVPAY  153
gi|225434984|spot 367|HSC70-2  KRLIGRRFSDSSVQSDIKLWPFKVIPGPGDKPMIVVVYKGEEKQFAAEEISSMVLIKMREIAEAYLGSTVKNAVVTVPAY  153
gi|147805297|spot 332|cpHSP70  KRFIGRKMD..EVGEEAKQVSYKVVRDENGN..VKLECPVLAKLFAAEEISAQVLRKLADDASKFLNDKVTKAVVTVPAY  156
consensus                      kr igr     v     k    kv                  k faaeeis  vl k    a   l   v  avvtvpay

gi|225449497|spot 355|HSC70-1  FNDSQRQATKDAGVIAGLNVMRIINEPTAAAIAYGLDKKATSVGEKNVLIFDLGGGTFDVSLLTIEEGIFEVKATAGDTH  233
gi|225449497|spot 361|HSC70-1  FNDSQRQATKDAGVIAGLNVMRIINEPTAAAIAYGLDKKATSVGEKNVLIFDLGGGTFDVSLLTIEEGIFEVKATAGDTH  233
gi|255573627|spot 358|HSC70-2  FNDSQRQATKDAGVIAGLNVMRIINEPTAAAIAYGLDKKATSVGEKNVLIFDLGGGTFDVSLLTIEEGIFEVKATAGDTH  233
gi|225434984|spot 367|HSC70-2  FNDSQRQATKDAGVIAGLNVMRIINEPTAAAIAYGLDKKATSVGEKNVLIFDLGGGTFDVSLLTIEEGIFEVKATAGDTH  233
gi|147805297|spot 332|cpHSP70  FNDSQRTATKDAGRIAGLEVLRIINEPTAASLAYGFEKKNN....ETILVFDLGGGTFDVSVLEVGDGVFEVLSTSGDTH  232
consensus                      fndsqr atkdag iagl v riineptaa  ayg  kk         l fdlgggtfdvs l    g fev  t gdth

gi|225449497|spot 355|HSC70-1  LGGEDFDNRMVNHFVQEFKRKHKKDITGNARSLRRLRTSCERAKRTLSSTAQTTIEI...DSLYEG.IDFYSTITRARFE  309
gi|225449497|spot 361|HSC70-1  LGGEDFDNRMVNHFVQEFKRKHKKDITGNARSLRRLRTSCERAKRTLSSTAQTTIEI...DSLYEG.IDFYSTITRARFE  309
gi|255573627|spot 358|HSC70-2  LGGEDFDNRMVNHFVQEFKRKNKKDISGNPRALRRLRTSCERAKRTLSSTAQTTIEI...DSLYEG.IDFYSTITRARFE  309
gi|225434984|spot 367|HSC70-2  LGGEDFDNRMVNHFVQEFKRKHKKDISGNPRALRRLRTSCERAKRTLSSTAQTTIEI...DSLYEG.IDFYSTITRARFE  309
gi|147805297|spot 332|cpHSP70  LGGDDFDKRIVDWLAQNFKRDEGIDLLKDKQALQRLTETAEKAKIELSSLTQTNISLPFITATSEGPKHIETTLTRAKFE  312
consensus                      lgg dfd r v    q fkr    d       l rl    e ak  lss  qt i         eg      t tra fe

gi|225449497|spot 355|HSC70-1  ELNMDLFRKCMEPVEKCLRDAKMDKSTIHDVVLVGGSTRIPKVQQLLQDFFNGKELCKSINPDEAVAYGAAVQAAILSGE  389
gi|225449497|spot 361|HSC70-1  ELNMDLFRKCMEPVEKCLRDAKMDKSTIHDVVLVGGSTRIPKVQQLLQDFFNGKELCKSINPDEAVAYGAAVQAAILSGE  389
gi|255573627|spot 358|HSC70-2  ELNMDLFRKCMEPVEKCLRDAKMDKSTVHDVVLVGGSTRIPKVQQLLQDFFNGKELCKSINPDEAVAYGAAVQAAILSGE  389
gi|225434984|spot 367|HSC70-2  ELNMDLFRKCMEPVEKCLRDAKMDKSTVHDVVLVGGSTRIPKVQQLLQDFFNGKELCKSINPDEAVAYGAAVQAAILSGE  389
gi|147805297|spot 332|cpHSP70  ELCSDLLDRLRTPVETALRDAKLSFKDLDEVILVGGSTRIPAVQDLVRKMT.GKEPNVTVNPDEVVALGAAVQAGVLAG.  390
consensus                      el  dl      pve  lrdak        v lvggstrip vq l      gke     npde va gaavqa  l g

gi|225449497|spot 355|HSC70-1  GNEKVQDLLLLDVTPLSLGLETAGGVMTVLIPRNTTIPTKKEQVFSTYSDNQPGVLIQVYEGERTRTRDNNLLGKFELSG  469
gi|225449497|spot 361|HSC70-1  GNEKVQDLLLLDVTPLSLGLETAGGVMTVLIPRNTTIPTKKEQVFSTYSDNQPGVLIQVYEGERTRTRDNNLLGKFELSG  469
gi|255573627|spot 358|HSC70-2  GNEKVQDLLLLDVTPLSLGLETAGGVMTVLIPRNTTIPTKKEQVFSTYSDNQPGVLIQVYEGERTRTRDNNLLGKFELSG  469
gi|225434984|spot 367|HSC70-2  GNEKVQDLLLLDVTPLSLGLETAGGVMTVLIPRNTTIPTKKEQVFSTYSDNQPGVLIQVYEGERTRTRDNNLLGKFELSG  469
gi|147805297|spot 332|cpHSP70  ...DVSNIVLLDVTPLSLGLETLGGVMTKIIPRNTTLPTSKSEVFSTAADGQTSVEINVLQGEREFVRDNKSLGSFRLDG  467
consensus                          v    lldvtplslglet ggvmt  iprntt pt k  vfst   d q  v i v ger   rdn  lg f l g

gi|225449497|spot 355|HSC70-1  IPPAPRGVPQITVCFDIDANGILNVSAEDKTTGQKNKITITNDKGRLSKEEIENMVQEAEKYKSEDEEHKKKVEAKNALE  549
gi|225449497|spot 361|HSC70-1  IPPAPRGVPQITVCFDIDANGILNVSAEDKTTGQKNKITITNDKGRLSKEEIENMVQEAEKYKSEDEEHKKKVEAKNALE  549
gi|255573627|spot 358|HSC70-2  IPPAPRGVPQITVCFDIDANGILNVSAEDKTTGQKNKITITNDKGRLSKEEIEKMVQEAEKYKSEDEEHKKKVEAKNALE  549
gi|225434984|spot 367|HSC70-2  IPPAPRGVPQITVCFDIDANGILNVSAEDKTTGQKNKITITNDKGRLSKEEIEKMVQEAEKYKSEDEEHKKKVEAKNALE  549
gi|147805297|spot 332|cpHSP70  IPPAPRGVPQIEVKFDIDANGILSVTAVDKGTGKKQDITITG.ASTLPNDEVDRMVKEAEKFAKEDKEKRDAIDTKNQAD  546
consensus                      ippaprgvpqi v fdidangil v a dk tg k  itit      l   e  mv eaek   ed e       kn

gi|225449497|spot 355|HSC70-1  NYAYNMRNTVKDEKISAKLPPADKKKIEDAVEQAIQWLDSNQLAEADEFEDKMKELESICNPIIAKMYQGAGGPDAGAGA  629
gi|225449497|spot 361|HSC70-1  NYAYNMRNTVKDEKISAKLPPADKKKIEDAVEQAIQWLDSNQLAEADEFEDKMKELESICNPIIAKMYQGAGGPDAGAGA  629
gi|255573627|spot 358|HSC70-2  NYAYNMRNTVKDEKIGAKLSPADKKKIEDAIESAIQWLDSNQLAEADEFEDKMKELESICNPIIAKMYQGAG.GEMGA.G  627
gi|225434984|spot 367|HSC70-2  NYAYNMRNTIKDEKIGAKLTPADKKKIEDAIDQAIQWLDSNQLAEADEFEDKMKELESICNPIIAKMYQGAG.PDMGG.P  627
gi|147805297|spot 332|cpHSP70  SVVYQTEKQLK..ELGDKVPAPVKGKVEAKLKELKDAISSG..STTQTIKDAMAALNQEVMQIGQSLYSQAGAPGAGAGA  622
consensus                         y      k      k     k k e           s          d m  l      i    y  ag    g

gi|225449497|spot 355|HSC70-1  MDDDGPSAGGSGAGPKIEEVD                                                             650
gi|225449497|spot 361|HSC70-1  MDDDGPSAGGSGAGPKIEEVD                                                             650
gi|255573627|spot 358|HSC70-2  MDEDAP.AGGSGAGPKIEEVD                                                             647
gi|225434984|spot 367|HSC70-2  MEDDVPPASGSGAGPKIEEVD                                                             648
gi|147805297|spot 332|cpHSP70  GAAGSTDSKPDGDVIDADFTES                                                      E      644
consensus                                 g
```

Figure S2. *Cont.*

```
gi|9294618|spot 170|PreP1  MSLTSSTAALRVPSRNLRRISSPSVAGRRLLLRRGLRIPSAAVRSVNGQFSRLSVRAVATQPAPLYPDVGQDEAEKLGFE    80
gi|9294618|spot 179|PreP1  MSLTSSTAALRVPSRNLRRISSPSVAGRRLLLRRGLRIPSAAVRSVNGQFSRLSVRAVATQPAPLYPDVGQDEAEKLGFE    80
consensus                  msltsstaalrvpsrnlrrisspsvagrrlllrrglripsaavrsvngqfsrlsvravatqpaplypdvgqdeaeklgfe

gi|9294618|spot 170|PreP1  KVSEEFISECKSKAILFKHKKTGCEVMSVSNEDENKVFGVVFRTPPKDSTGIPHILEHSVLCGSRKYPVKEPFVELLKGS   160
gi|9294618|spot 179|PreP1  KVSEEFISECKSKAILFKHKKTGCEVMSVSNEDENKVFGVVFRTPPKDSTGIPHILEHSVLCGSRKYPVKEPFVELLKGS   160
consensus                  kvseefiseckskailfkhkktgcevmsvsnedenkvfgvvfrtppkdstgiphilehsvlcgsrkypvkepfvellkgs

gi|9294618|spot 170|PreP1  LHTFLNAFTYPDRTCYPVASTNTKDFYNLVDVYLDAVFFPKCVDDAHTFQQEGWHYELNDPSEDISYKGVVFNEMKGVYS   240
gi|9294618|spot 179|PreP1  LHTFLNAFTYPDRTCYPVASTNTKDFYNLVDVYLDAVFFPKCVDDAHTFQQEGWHYELNDPSEDISYKGVVFNEMKGVYS   240
consensus                  lhtflnaftypdrtcypvastntkdfynlvdvyldavffpkcvddahtfqqegwhyelndpsedisykgvvfnemkgvys

gi|9294618|spot 170|PreP1  QPDNILGRIAQQALSPENTYGVDSGGDPKDIPNLTFEEFKEFHRQYYHPSNARIWFYGDDDPVHRLRVLSEYLDMFEASP   320
gi|9294618|spot 179|PreP1  QPDNILGRIAQQALSPENTYGVDSGGDPKDIPNLTFEEFKEFHRQYYHPSNARIWFYGDDDPVHRLRVLSEYLDMFEASP   320
consensus                  qpdnilgriaqqalspentygvdsggdpkdipnltfeefkefhrqyyhpsnariwfygdddpvhrlrvlseyldmfeasp

gi|9294618|spot 170|PreP1  SPNSSKIKFQKLFSEPVRLVEKYPAGRDGDLKKKHMLCVNWLLSEKPLDLQTQLALGFLDHLMLGTPASPLRKILLESGL   400
gi|9294618|spot 179|PreP1  SPNSSKIKFQKLFSEPVRLVEKYPAGRDGDLKKKHMLCVNWLLSEKPLDLQTQLALGFLDHLMLGTPASPLRKILLESGL   400
consensus                  spnsskikfqklfsepvrlvekypagrdgdlkkkhmlcvnwllsekpldlqtqlalgfldhlmlgtpasplrkillesgl

gi|9294618|spot 170|PreP1  GEALVSSGLSDELLQPQFGIGLKGVSEENVQKVEELIMDTLKKLAEEGFDNDAVEASMNTIEFSLRENNTGSFPRGLSLM   480
gi|9294618|spot 179|PreP1  GEALVSSGLSDELLQPQFGIGLKGVSEENVQKVEELIMDTLKKLAEEGFDNDAVEASMNTIEFSLRENNTGSFPRGLSLM   480
consensus                  gealvssglsdellqpqfgiglkgvseenvqkveelimdtlkklaeegfdndaveasmntiefslrenntgsfprglslm

gi|9294618|spot 170|PreP1  LQSISKWIYDMDPFEPLKYTEPLKALKTRIAEEGSKAVFSPLIEKLILNNSHRVTIEMQPDPEKATQEEVEEKNILEKVK   560
gi|9294618|spot 179|PreP1  LQSISKWIYDMDPFEPLKYTEPLKALKTRIAEEGSKAVFSPLIEKLILNNSHRVTIEMQPDPEKATQEEVEEKNILEKVK   560
consensus                  lqsiskwiydmdpfeplkyteplkalktriaeegskavfsplieklilnnshrvtiemqpdpekatqeeveeknilekvk

gi|9294618|spot 170|PreP1  AAMTEEDLAELARATEELKLKQETPDPPEALRCVPSLNLGDIPKEPTYVPTEVGDINGVKVLRHDLFTNDIIYTEVVFDI   640
gi|9294618|spot 179|PreP1  AAMTEEDLAELARATEELKLKQETPDPPEALRCVPSLNLGDIPKEPTYVPTEVGDINGVKVLRHDLFTNDIIYTEVVFDI   640
consensus                  aamteedlaelarateelklkqetpdppealrcvpslnlgdipkeptyvptevgdingvkvlrhdlftndiiytevvfdi

gi|9294618|spot 170|PreP1  GSLKHELLPLVPLFCQSLLEMGTKDLTFVQLNQLIGRKTGGISVYPLTSSVRGKDEPCSKIIVRGKSMAGRADDLFNLMN   720
gi|9294618|spot 179|PreP1  GSLKHELLPLVPLFCQSLLEMGTKDLTFVQLNQLIGRKTGGISVYPLTSSVRGKDEPCSKIIVRGKSMAGRADDLFNLMN   720
consensus                  gslkhellplvplfcqsllemgtkdltfvqlnqligrktggisvypltssvrgkdepcskiivrgksmagraddlfnlmn

gi|9294618|spot 170|PreP1  CLLQEVQFTDQQRFKQFVSQSRARMENRLRGSGHGIAAARMDAMLNIAGWMSEQMGGLSYLEFLHTLEKKVDEDWEGISS   800
gi|9294618|spot 179|PreP1  CLLQEVQFTDQQRFKQFVSQSRARMENRLRGSGHGIAAARMDAMLNIAGWMSEQMGGLSYLEFLHTLEKKVDEDWEGISS   800
consensus                  cllqevqftdqqrfkqfvsqsrarmenrlrgsghgiaaarmdamlniagwmseqmgglsyleflhtlekkvdedwegiss

gi|9294618|spot 170|PreP1  SLEEIRRSLLARNGCIVNMTADGKSLTNVEKSVAKFLDLLPENPSGGLVTWDGRLPLRNEAIVIPTQVNYVGKAGNIYST   880
gi|9294618|spot 179|PreP1  SLEEIRRSLLARNGCIVNMTADGKSLTNVEKSVAKFLDLLPENPSGGLVTWDGRLPLRNEAIVIPTQVNYVGKAGNIYST   880
consensus                  sleeirrsllarngcivnmtadgksltnveksvakfldllpenpsgglvtwdgrlplrneaiviptqvnyvgkagniyst

gi|9294618|spot 170|PreP1  GYELDGSAYVISKHISNTWLWDRVRVSGGAYGGFCDFDSHSGVFSYLSYRDPNLLKTLDIYDGTGDFLRGLDVDQETLTK   960
gi|9294618|spot 179|PreP1  GYELDGSAYVISKHISNTWLWDRVRVSGGAYGGFCDFDSHSGVFSYLSYRDPNLLKTLDIYDGTGDFLRGLDVDQETLTK   960
consensus                  gyeldgsayviskhisntwlwdrvrvsggayggfcdfdshsgvfsylsyrdpnllktldiydgtgdflrgldvdqetltk

gi|9294618|spot 170|PreP1  AIIGTIGDVDSYQLPDAKGYSSLLRHLLGVTDEERQRKREEILTTSLKDFKDFAQAIDVVRDKGVAVAVASAEDIDAANN  1040
gi|9294618|spot 179|PreP1  AIIGTIGDVDSYQLPDAKGYSSLLRHLLGVTDEERQRKREEILTTSLKDFKDFAQAIDVVRDKGVAVAVASAEDIDAANN  1040
consensus                  aiigtigdvdsyqlpdakgyssllrhllgvtdeerqrkreeilttslkdfkdfaqaidvvrdkgvavavasaedidaann

gi|9294618|spot 170|PreP1  ERSNFFEVKKA                                                                      1051
gi|9294618|spot 179|PreP1  ERSNFFEVKKA                                                                      1051
consensus                  ersnffevkka
```

F

Figure S2. Multiple alignment of the amino acid sequences from proteoforms by using DNAMAN software (Lynnon Corporation, QC, Canada). (**A**) glutamine synthetase isoform GS1c (GS1), with 100% identities; (**B**) transketolase (TK), with 84.41% identities; (**C**) RuBisCO large subunit-binding protein subunit (RBP), with 72.68% identities; (**D**) phosphoribulokinase (PRK), with 80.64% identities; (**E**) heat shock protein 70 (Hsp70), with 86.09% identities; (**F**) zinc metalloprotease (insulinase family), homologous to presequence protease 1 (PreP1), with 100% identities. The gi number, spot number, and protein name of the presented proteins are shown on the left.

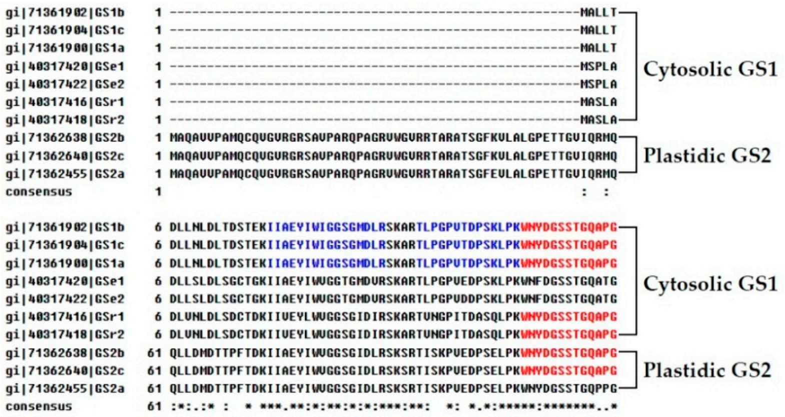

Figure S3. *Cont.*

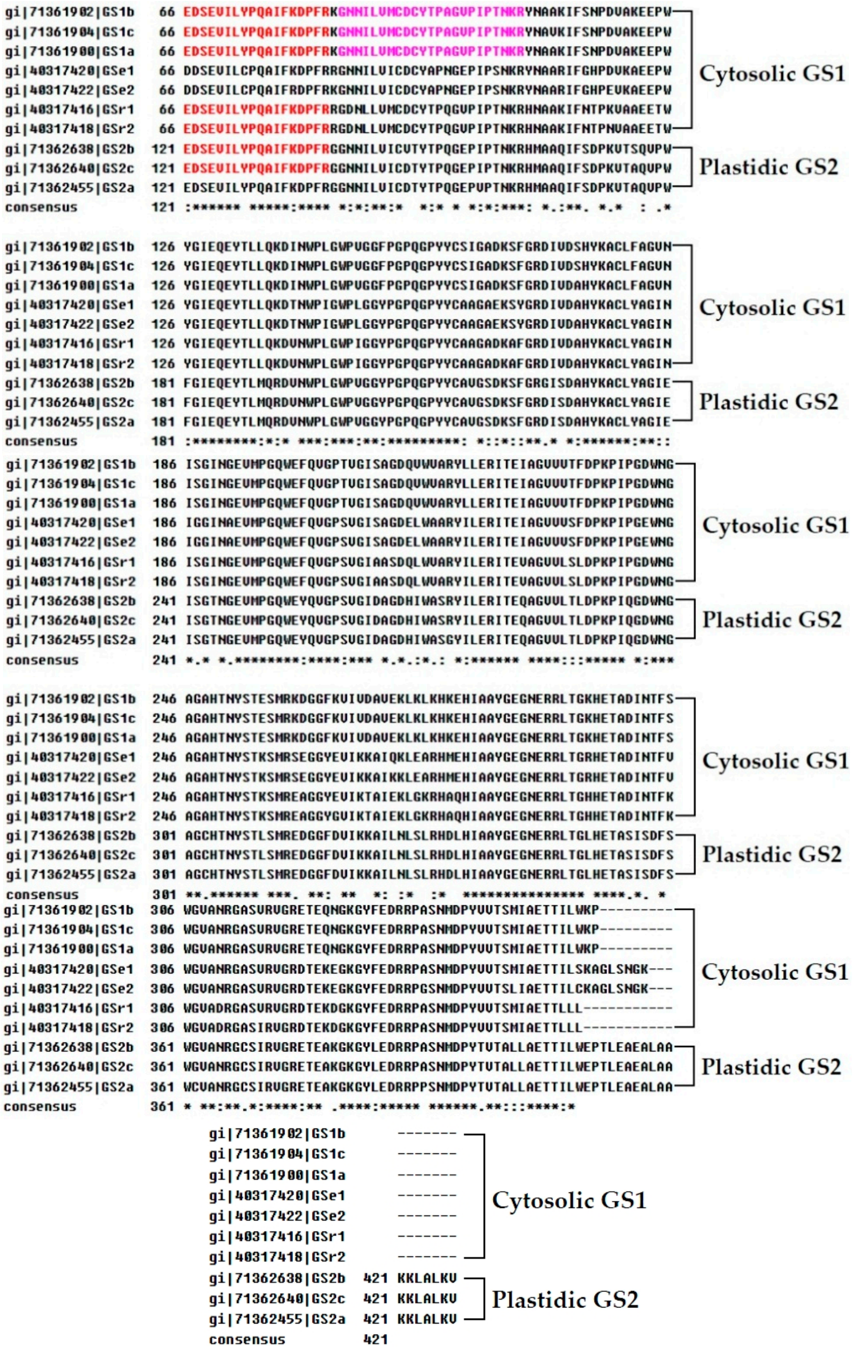

Figure S3. The alignment analysis between the MS-identified peptides from two proteoforms of GS1 (spots 795 and 806) and the amino acid sequences of the ten glutamine synthetase (GS) isozymes in wheat (*Triticum aestivum* L.). The amino acid sequences from wheat used for the multiple sequence alignment werefrom NCBI protein database (http://www.ncbi.nlm.nih.gov/protein/) and submitted by Bernard *et al*. [36]. The accession numbers and names of the GS isozymes were listed on the left, and the subfamilies were shown on the right of the amino acid sequences. The asterisk indicates completely conserved residues; colon indicates highly conserved residues; dot indicates a weak conservative region. The MS-identified peptides from spots 795, 806 and both were highlighted in red, blue, and purple fonts, respectively. The results showed that the MS-identified peptides were well aligned with cytosolic GS1, but not plastidic GS2. The detailed information of the identified peptides can be found in Table S1.

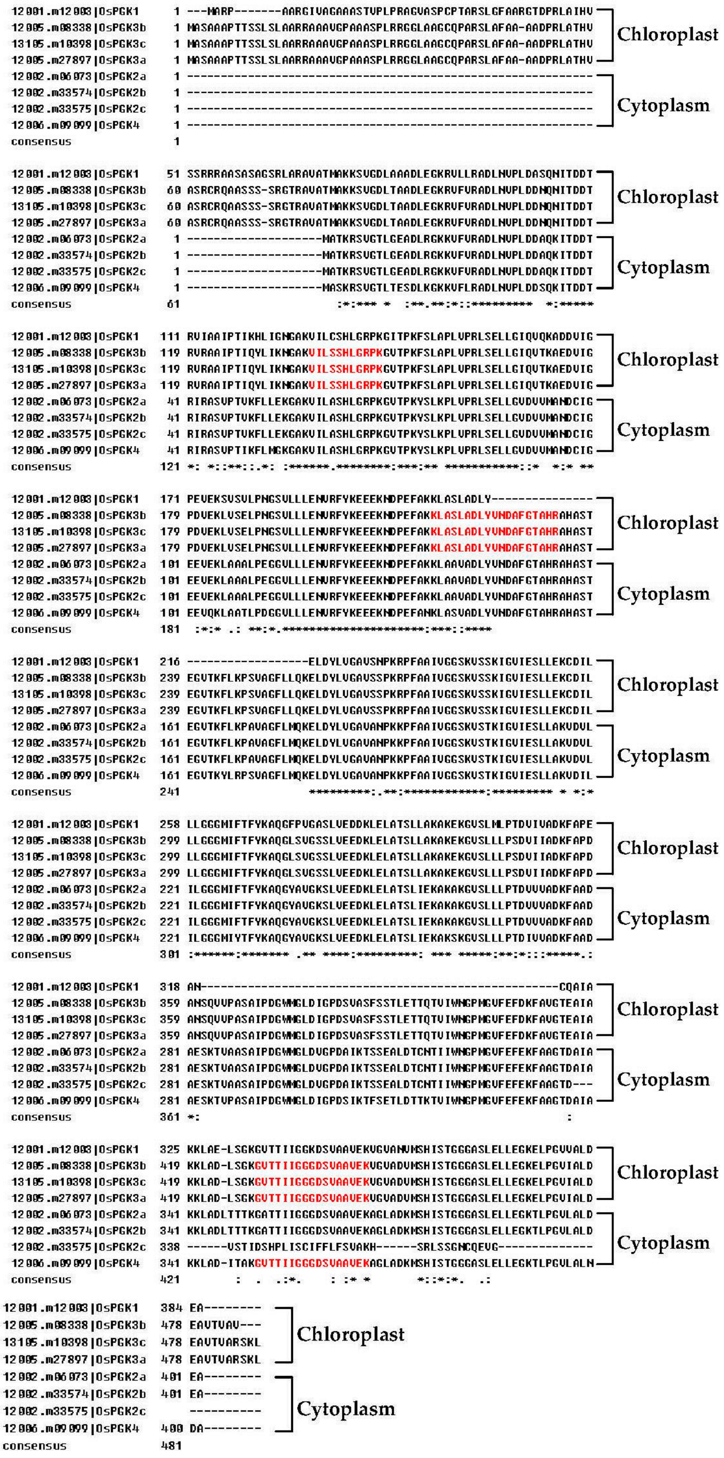

Figure S4. The alignment analysis between the MS-identified peptides of phosphoglycerate kinase (PGK) from *Ricinus communis* and the amino acid sequences of the eight putative protein products encoded from four Pgk genes in *Oryza sativa* sp. Japonica. The amino acid sequences from *O. sativa* sp. Japonica used for the multiple sequence alignment were from GABI primary database (https://www.gabipd.org) by using the TIGR ID provided by Joshi *et al*. [37].The detailed accession numbers and names of the PGK isozymes were listed on the left, and the subcellular locations were shown on the right of the amino acid sequences. The asterisk indicates completely conserved residues; colon indicates highly conserved residues; dot indicates a weak conservative region; the short line indicates the vacancies residues of optimal sequence alignment. The identified peptides of PGK identified from *Ricinus communis* were highlighted in red fonts. The results showed that the unique matched peptides could only be found in the three members of OsPGK3, which were predicted to be localized in chloroplast. The detailed information of the identified peptides can be found in Table S1.

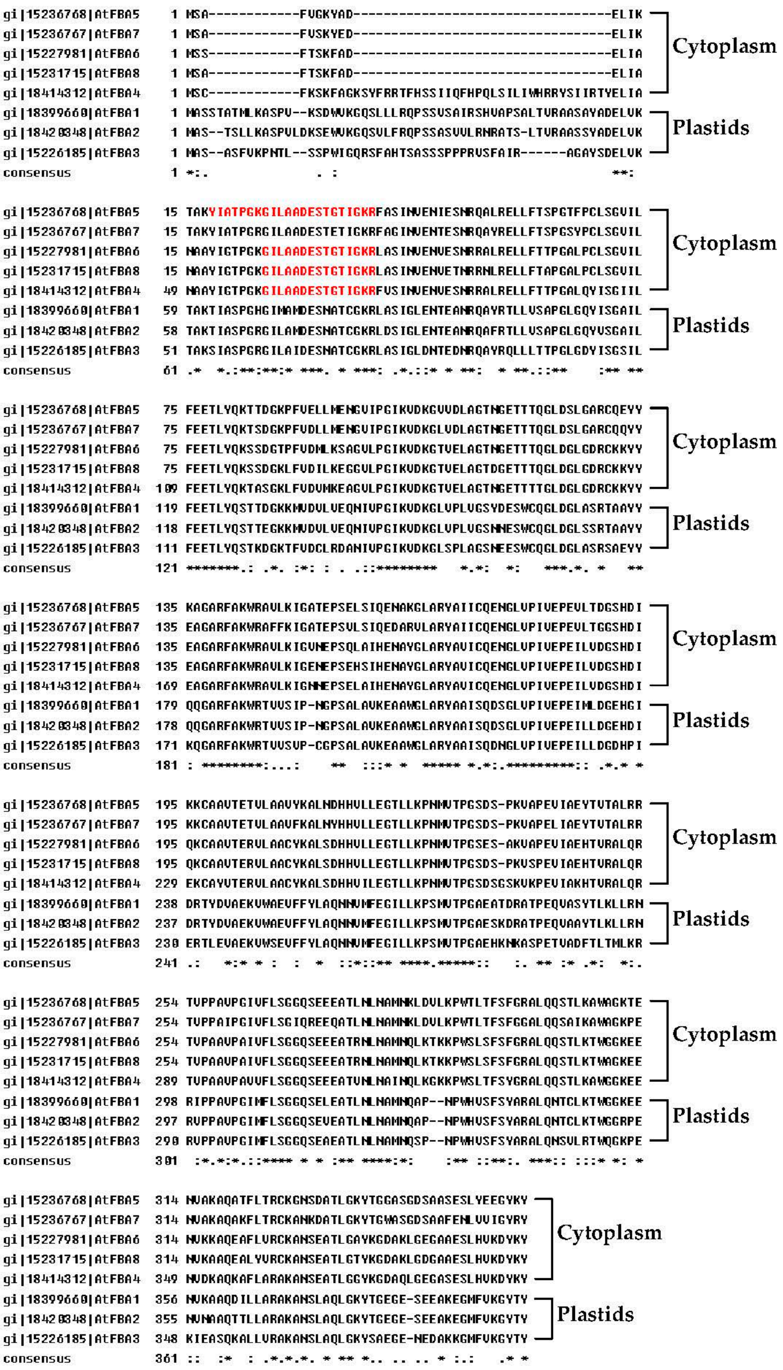

Figure S5. The alignment analysis between the MS-identified peptides of fructose 1,6-biphosphate aldolase (FBA) from *Pandanus amaryllifolius* and the amino acid sequences of the eight FBA family genes (AtFBA1-8) encoded proteins in *Arabidopsis thaliana*. The amino acid sequences from *Arabidopsis thaliana* used for the multiple sequence alignment were from NCBI protein database (http://www.ncbi.nlm.nih.gov/protein/) by using the protein ID provided by Lu *et al*. [38]. The detailed accession numbers and names of the FBA isozymes were listed on the left, and the subcellular localizations were shown on the right of the amino acid sequences. The asterisk indicates completely conserved residues; colon indicates highly conserved residues; dot indicates a weak conservative region; the short line indicates the vacancies residues of optimal sequence alignment. The identified peptides of FBA identified from *Pandanus amaryllifolius* were highlighted in red fonts. The results showed that the matched peptides can only be found in the four FBAs (AtFBA4, 5, 6, 8) that localized in the cytoplasm, and none of matched peptides can be found in the FBAs (AtFBA1-3) that localized in the plastids. The detailed information of the identified peptides can be found in Table S1.

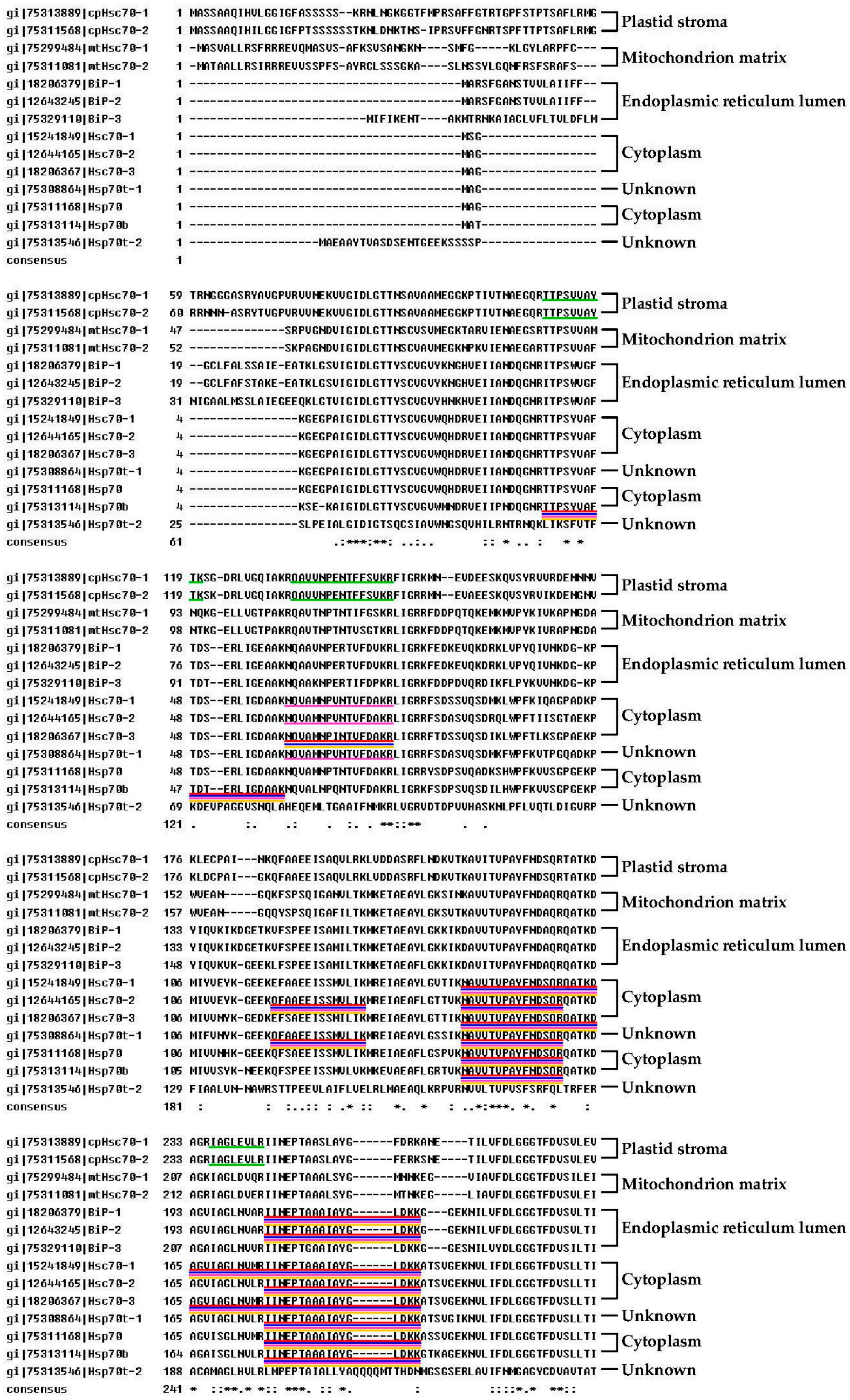

Figure S6. *Cont.*

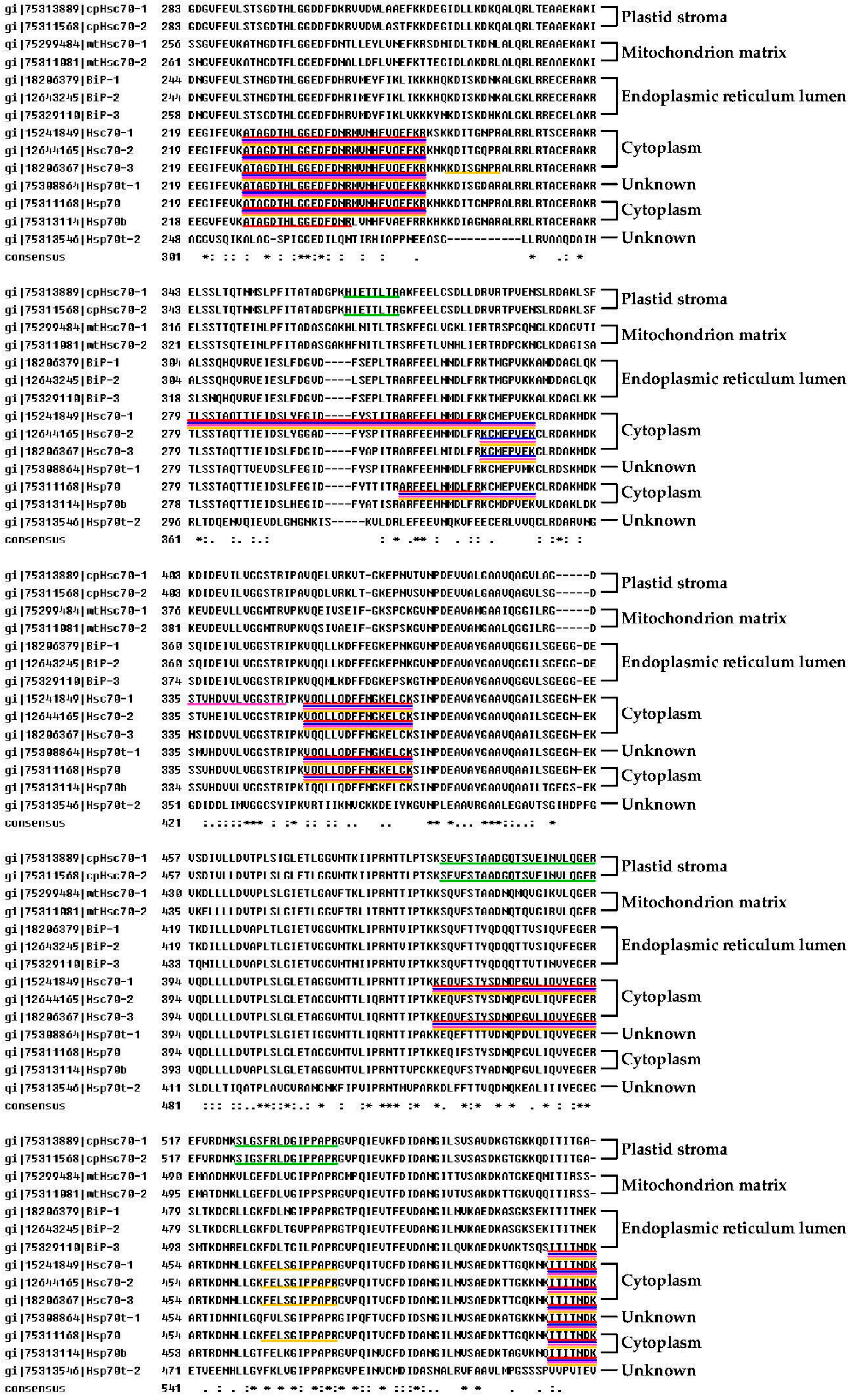

Figure S6. *Cont.*

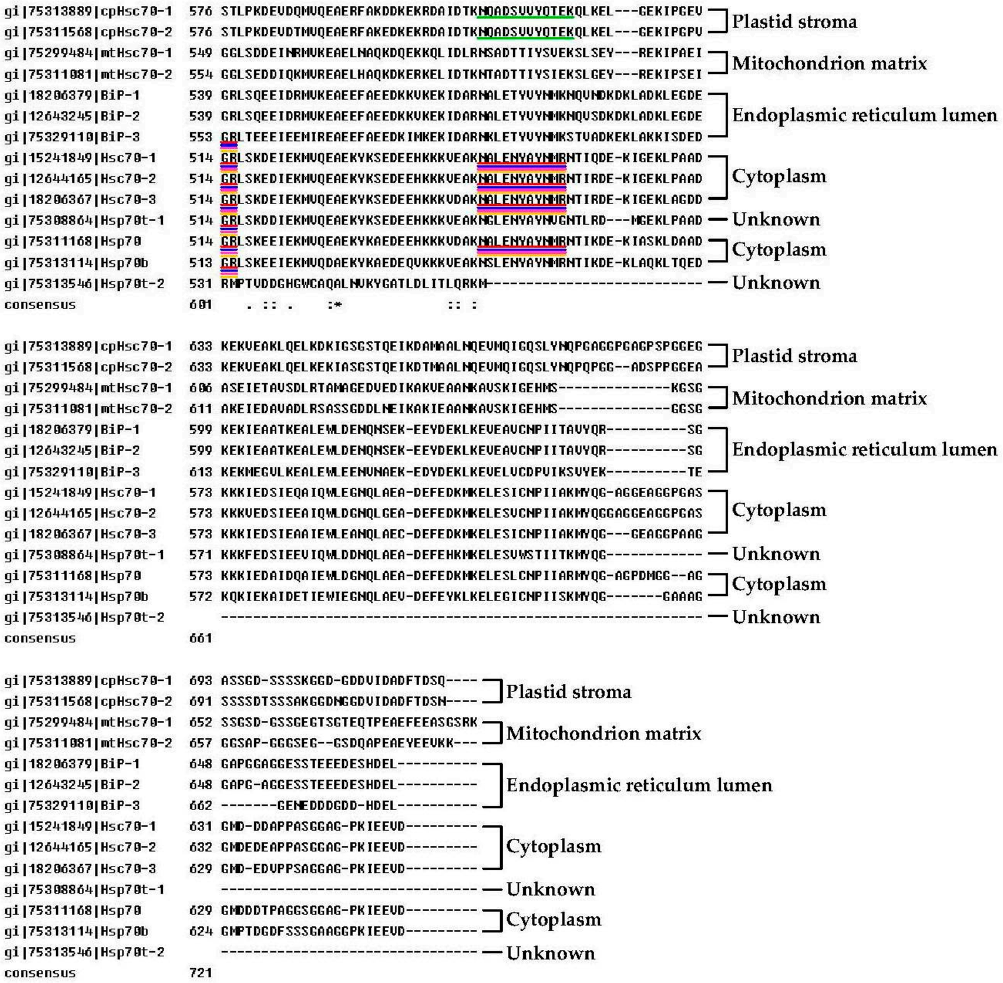

Figure S6. The alignment analysis between the MS-identified peptides from five proteoforms of heat shock protein 70 (Hsp70) (spots 355, 361, 358, 367, and 332) and the amino acid sequences of the fourteen Hsp70 gene family encoded proteins in Arabidopsis. The amino acid sequences from Arabidopsis used for the multiple sequence alignment were from NCBI protein database (http://www.ncbi.nlm.nih.gov/protein/) by using the protein name provided by Sung *et al*. [39]. The detailed accession numbers and names of the HSP70 proteins were listed on the left, and the subcellular locations were shown on the right of the amino acid sequences. The asterisk indicates completely conserved residues; colon indicates highly conserved residues; dot indicates a weak conservative region; the short line indicates the vacancies residues of optimal sequence alignment. The identified peptides from spots 355, 361, 358, 367, and 332 were highlighted with red, blue, purple, yellow, and green underlines, respectively. The results showed that the unique identified peptides from all the spots 355, 361, 358, and 367 could be only matched to the Hsp70s localized in cytoplasm. In contrast, the unique identified peptides from the spot 332 could be only matched to the Hsp70s localized in chloroplast. The detailed information of the identified peptides can be found in Table S1.

Table S3. Proteoforms identified in *Hosta* "Gold standard".

Protein Name [a]	Identity [b]	Spot No. [c]	SCL [d]	Plant Species [e]	gi Number [f]	Exp. *M*w (Da)/pI [g]	Thr. *M*w (Da)/pI [h]	Sco [i]	Cov (%) [j]	QM [k]	V% ± SE [l] YLM MLM LENF YLC MLC
glutamine synthetase isoform GS1c (GS1)	100%	795	Cyt	*Triticum aestivum*	71361904	39,676/5.58	39,445/5.41	89	15	2	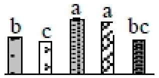
		806	Cyt	*T. aestivum*	71361904	39,505/5.75	39,445/5.41	168	15	3	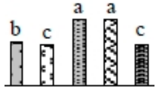
transketolase (TK)	84.41%	338	Chl	*Nicotiana tabacum*	194396261	78,879/5.95	80,451/6.16	141	17	10	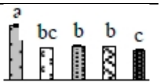
		346	Chl	*Populus trichocarpa*	224063766	79,837/5.87	81,122/5.97	337	13	9	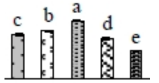
RuBisCO large subunit-binding protein subunit (RBP)	72.68%	476	Chl	*Vitis vinifera*	225442531	63,140/5.18	65,255/5.6	474	17	8	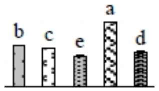
		459	Chl	*Sorghum bicolor*	242090109	66,151/4.84	60,914/5.07	233	31	13	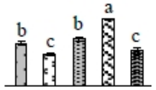
		472	Chl	*Zea mays*	223948025	64,795/5.24	61,969/5.42	524	30	11	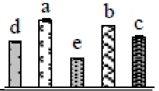
phosphoribulokinase (PRK)	80.64%	840	Chl	*Pisum sativum*	1885326	38,189/4.99	39,230/5.41	113	13	3	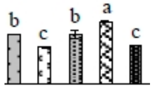
		832	Chl	*Glycine max*	255646270	38,858/4.85	45,757/5.89	277	38	11	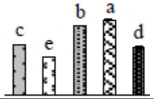

Table S1. *Cont.*

Protein Name [a]	Identity [b]	Spot No. [c]	SCL [d]	Plant Species [e]	gi Number [f]	Exp. *Mw* (Da)/pI [g]	Thr. *Mw* (Da)/pI [h]	Sco [i]	Cov (%) [j]	QM [k]	V% ± SE [l] YLM MLM LENF YLC MLC
heat shock protein 70 kDa (Hsp70)	86.09%	355	Cyt	*V. vinifera*	225449497	77,129/5.15	71,525/5.17	1050	61	26	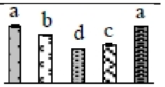
		361	Cyt	*V. vinifera*	225449497	77,663/5.10	71,525/5.17	959	58	25	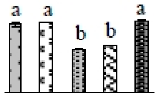
		358	Cyt	*Ricinus communis*	255573627	78,743/5.06	71,276/5.14	977	53	23	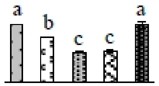
		367	Cyt	*V. vinifera*	225434984	76,864/5.20	71,589/5.17	847	57	25	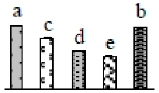
		332	Chl	*V. vinifera*	147805297	80,390/4.75	69,198/5.11	722	36	16	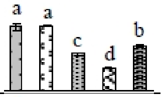
zinc metalloprotease (insulinase family), homologous to presequence protease 1 (PreP1) *	100%	170	Chl/Mit	*Arabidopsis thaliana*	9294618	113,711/4.99	118,265/5.32	346	13	11	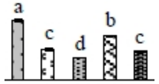
		179	Chl/Mit	*A. thaliana*	9294618	113,515/5.07	118,265/5.32	157	11	9	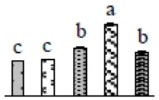

[a]: The name and functional categories of the proteins identified by MALDI TOF-TOF MS. Protein names marked with an asterisk (*) have been edited by us depending on BLAST against NCBI non-redundant protein database. The detailed information of the NCBI BLAST can be found in Table S2; [b]: The identities of the multiple alignment of the amino acid sequences from different proteoforms. [c]: Assigned spot number as indicated in Figure 5; [d]: Protein subcellular localization predicted by softwares (YLoc, LocTree3, Plant-mPLoc, ngLOC, and TargetP). SCL, subcellular location; Chl, chloroplast; Cyt, cytoplasm; Mit, mitochondria; [e]: The plant species that the peptides matched from. [f]: Database accession numbers from NCBInr; [g,h]: Experimental (f) and theoretical (g) molecular weight (Da) and pI of identified proteins. Experimental values were calculated using Image Master 2D Platinum Software. Theoretical values were retrieved from the protein database; [i]: The Mascot score obtained after searching against the NCBInr database; [j]: The amino acid sequence coverage for the identified proteins; [k]: The number of unique peptides identified for each protein; [l]: The mean values of protein spot volumes relative to total volume of all the spots. Five samples (from left to right: YLM, YLC, MLM, MLC, LENF) of various leaf regions in different development stages of *Hosta* "Gold Standard" were performed. YLM, young leaf margin; YLC, young leaf center; MLM, mature leaf margin; MLC, mature leaf center; LENF, leaf under excess nitrogen fertilization. The different small letters on the columns indicate significant differences ($p < 0.05$) among the five samples as determined by one-way ANOVA. Error bars indicate ± standard error (SE).
